# Perinatal HPA axis regulation and offspring neuropsychiatric development: mechanisms, developmental trajectories, and clinical implications

**DOI:** 10.3389/fendo.2026.1922174

**Published:** 2026-08-28

**Authors:** Yanwei Guo, Jinyi Liu, Yaru Peng, Xiaofeng Li

**Affiliations:** 1 Department of Reproductive Medicine, Hebei Maternity Hospital, Shijiazhuang, Hebei, China; 2 Division of Psychology and Language Sciences, Faculty of Brain Sciences, University College London, London, United Kingdom; 3 Department of Gynaecological Oncology, Gansu Provincial Cancer Hospital, Lanzhou, Gansu, China; 4 Department of Women and Children’s Health, School of Life Course Sciences, King’s College London, London, United Kingdom

**Keywords:** antenatal corticosteroids, developmental origins of health and disease, maternal hypothalamic–pituitary–adrenal axis, offspring neurodevelopment, placental glucocorticoid regulation, prenatal psychological distress

## Abstract

Maternal hypothalamic–pituitary–adrenal (HPA) axis regulation forms part of the maternal–placental–foetal system through which prenatal environments may influence offspring developmental trajectories. During pregnancy, cortisol supports placental function and foetal maturation; however, its developmental significance depends on exposure timing, magnitude, and duration, placental glucocorticoid regulation, and foetal susceptibility. This narrative review synthesises evidence regarding maternal psychological distress, endogenous glucocorticoid regulation, placental glucocorticoid pathways, antenatal synthetic glucocorticoid exposure, and offspring neuropsychiatric development. Human studies indicate that maternal psychological distress and HPA-axis-related measures represent related but distinct dimensions of prenatal exposure. Associations have been reported between depressive and anxiety symptoms, maternal cortisol profiles, placental HSD11B2/11β-HSD2-related measures, and offspring glucocorticoid regulation. However, findings vary according to exposure definition, gestational timing, biological matrix, placental sampling, foetal sex, and covariate adjustment. Most evidence remains observational and does not establish a continuous causal pathway from maternal psychological distress through placental glucocorticoid dysregulation to later neurodevelopmental outcomes. Across foetal life, infancy, and childhood, studies have examined associations with stress-system regulation, cognitive and language development, emotional and behavioural functioning, and psychiatric vulnerability. Mechanistic research implicates epigenetic, immune-inflammatory, neurotrophic, cellular, and microbial pathways, although their mediating roles in humans remain incompletely established. Antenatal corticosteroids provide substantial neonatal benefits when clinically indicated for threatened preterm birth; current uncertainty mainly concerns potential long-term effects of repeated or additional exposure. Psychosocial and dietary interventions may improve maternal wellbeing and selected physiological outcomes, but whether these effects are mediated through cortisol regulation remains unclear. Future research should integrate repeated endocrine phenotyping, placental functional assessment, early-life developmental phenotyping, causal-inference approaches, multi-omics strategies, and longitudinal developmental follow-up to distinguish causal mechanisms from adaptation, confounding, and correlated biomarkers.

## Highlights

- Maternal psychological distress and cortisol regulation are related but non-equivalent exposures.
- Placental glucocorticoid regulation varies according to gestational timing, tissue compartment, foetal sex, and biological context.
- Associations with offspring HPA-axis function and neurodevelopment differ across developmental stages and measurement approaches.
- Current interventions improve selected maternal outcomes, but cortisol-mediated prevention of offspring developmental risk has not been established.

## 1 Introduction

The perinatal period represents a critical developmental window characterised by maternal physiological adaptation, dynamic placental changes, and rapid neurodevelopmental processes in the offspring. During this stage, the foetal brain undergoes extensive structural and functional maturation, while maternal endocrine, immune, and placental systems adapt to support pregnancy and development (1, 2). Maternal psychological distress, including chronic stress, anxiety, and depressive symptoms, has been associated with altered offspring developmental trajectories involving cognitive, emotional, and behavioural domains (2, 3). However, psychological distress reflects subjective psychological experiences, whereas maternal hypothalamic–pituitary–adrenal (HPA) axis activity and cortisol secretion represent complex endocrine processes involved in both stress responses and normal pregnancy adaptation. Although these systems interact, psychological distress and cortisol should not be considered equivalent indicators of the same biological process (1, 4). The mechanisms linking maternal psychological states and HPA-axis regulation with offspring developmental trajectories remain incompletely understood (2, 5).

The developmental origins of health and disease (DOHaD) framework proposes that environmental exposures during sensitive developmental periods may influence later health through interacting endocrine, immune, metabolic, and epigenetic pathways (6–8). Developmental programming refers to persistent functional alterations induced by environmental influences during critical developmental windows (9). However, demonstrating developmental programming in humans remains challenging because most evidence is derived from observational studies, which identify associations but do not establish causal mechanisms. Therefore, findings regarding maternal HPA-axis regulation and cortisol should be interpreted as consistent with developmental programming hypotheses unless supported by experimental or quasi-experimental evidence (2, 5, 10).

Previous studies have examined associations between maternal HPA-axis regulation or cortisol concentrations and offspring outcomes, including stress-system regulation, emotional and behavioural functioning, cognitive and language development, and later neuropsychiatric vulnerability (11). Increasing evidence suggests that placental glucocorticoid metabolism, epigenetic regulation, immune-inflammatory pathways, and foetal sex-specific adaptation may contribute to variation in maternal–placental–foetal signalling and developmental susceptibility (12–14). Postnatal caregiving environments may further modify or buffer these developmental associations (15).

Recent reviews have provided important insights into prenatal stress, maternal HPA-axis adaptation, placental glucocorticoid regulation, and offspring outcomes. However, these components have often been examined separately rather than within an integrated developmental framework spanning pregnancy, birth, infancy, and childhood (2, 11, 16, 17). Furthermore, maternal cortisol cannot be considered a direct biological proxy for psychological stress, as cortisol regulation is influenced by gestational timing, placental function, metabolic status, and broader maternal and foetal contexts (1, 4, 18). The developmental significance of maternal HPA-axis regulation therefore requires interpretation within a dynamic maternal–placental–foetal system rather than through a single biomarker or exposure model.

Therefore, this review aims to provide an integrated framework linking maternal HPA-axis regulation, placental glucocorticoid signalling, developmental trajectories, and potential clinical implications.

Specifically, this review focuses on four major aspects: (1) the relationship between maternal psychological stress, anxiety, depression, and HPA-axis regulation, with emphasis on distinguishing psychological states from endocrine measures; (2) placental glucocorticoid regulation and related molecular mechanisms, including 11β-hydroxysteroid dehydrogenase type 2 (11β-HSD2), epigenetic regulation, immune-inflammatory pathways, and sex-specific adaptation; (3) associations between maternal HPA-axis alterations and offspring outcomes across developmental stages, including foetal life, neonatal period, infancy, and childhood, with emphasis on stress-system regulation, neuropsychiatric outcomes, and cognitive development; and (4) potential preventive and intervention strategies, together with current limitations of available evidence.

By integrating evidence from human cohort studies, clinical investigations, and experimental models, this review aims to clarify how maternal HPA-axis regulation contributes to maternal–foetal communication and offspring developmental trajectories, while identifying key questions for future research.

**Figure 1:**
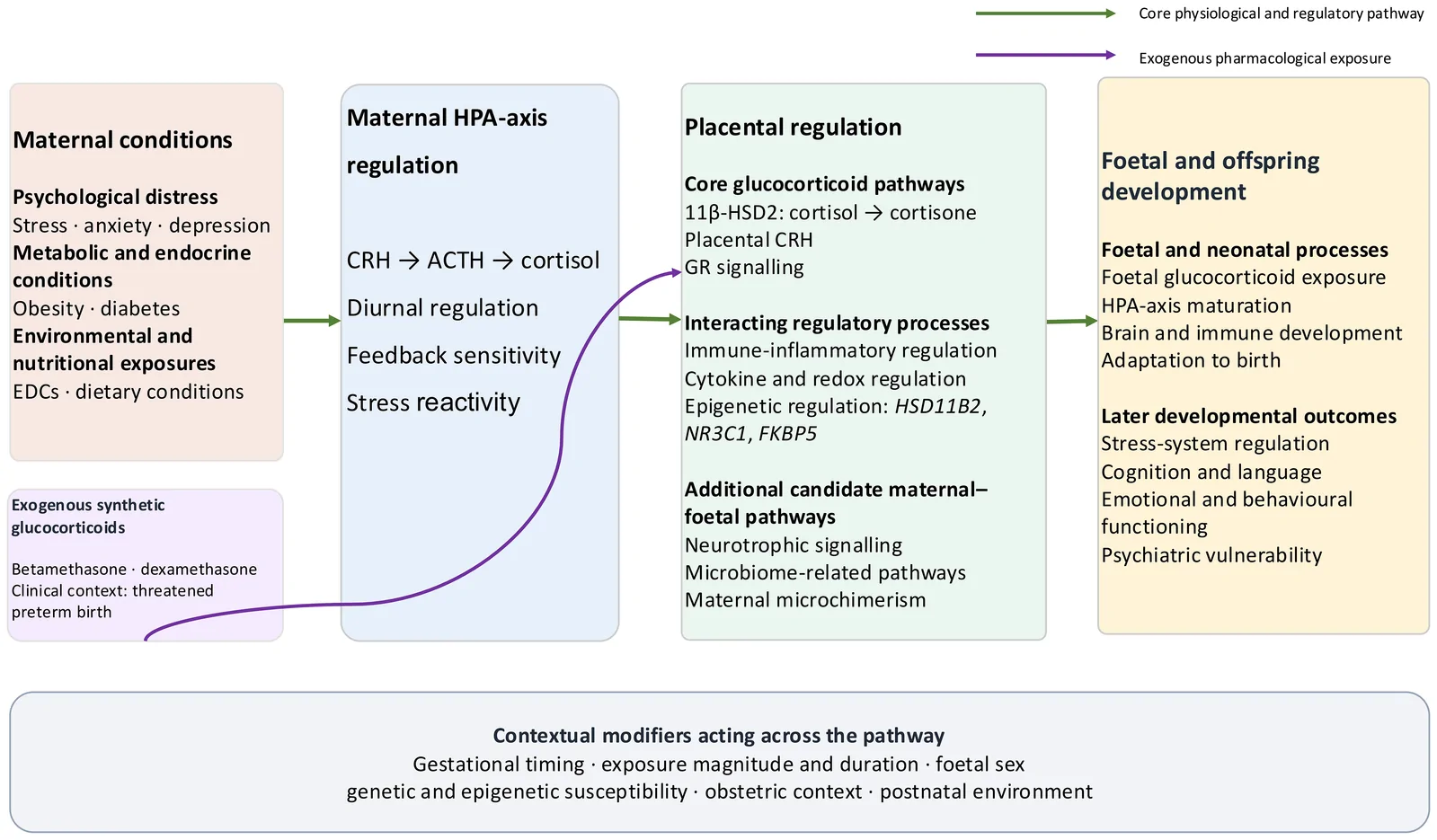
Integrated maternal–placental–fetal glucocorticoid signalling network and developmental pathways. Maternal psychological, metabolic, endocrine, nutritional, and environmental conditions may be associated with variation in maternal HPA-axis regulation and placental glucocorticoid signalling. The placenta regulates foetal glucocorticoid exposure through 11β-HSD2-mediated conversion of cortisol to cortisone, placental CRH, GR signalling, and interacting immune-inflammatory, redox, epigenetic, neurotrophic, microbial, and cellular processes. These pathways may contribute to foetal HPA-axis maturation, brain and immune development, adaptation to birth, and later stress regulation, cognitive and language development, emotional and behavioural functioning, and psychiatric vulnerability. Synthetic glucocorticoids represent a distinct exogenous pharmacological exposure. Green arrows indicate core physiological and regulatory pathways, whereas the purple arrow indicates exogenous pharmacological exposure. Associations across the framework are modified by gestational timing, exposure magnitude and duration, foetal sex, genetic and epigenetic susceptibility, obstetric context, and the postnatal environment. The figure represents an integrative conceptual framework rather than a single established causal sequence.

**Figure 1** summarises the integrated maternal–placental–foetal glucocorticoid signalling framework used to organise the evidence reviewed below.

## 2 Methods

A narrative literature review was conducted to summarise recent advances in maternal HPA-axis regulation, placental glucocorticoid signalling, and offspring developmental outcomes. Literature searches were performed in PubMed/MEDLINE from January 2019 to April 2026 using combinations of the following keywords: “maternal HPA axis”, “cortisol”, “glucocorticoid”, “pregnancy”, “prenatal stress”, “placenta”, “11β-HSD2”, “offspring”, “neurodevelopment”, “cognition”, “behavior”, “behaviour”, and “psychiatric outcomes”.

Recent human studies were prioritised; however, key earlier studies and experimental animal studies were included when they provided essential mechanistic evidence regarding HPA-axis regulation, placental glucocorticoid metabolism, epigenetic mechanisms, immune pathways, or developmental programming.

Studies were selected based on their relevance to the following domains: (1) physiological regulation of the maternal HPA axis during pregnancy; (2) psychological distress and endocrine alterations; (3) placental glucocorticoid signalling and foetal exposure; (4) offspring neurodevelopmental trajectories; and (5) potential preventive and therapeutic strategies.

As this was a narrative review, no formal meta-analysis or systematic quality assessment was performed.

## 3 Maternal HPA axis regulation during pregnancy

Glucocorticoids are steroid hormones produced by the adrenal cortex, with cortisol representing the predominant endogenous glucocorticoid in humans (1). Cortisol contributes to metabolic regulation, immune adaptation, stress responses, placental function, foetal maturation, and neurodevelopment during pregnancy (1, 19, 20). The maternal HPA axis, comprising hypothalamic CRH, pituitary ACTH, and adrenal cortisol secretion, is the principal endocrine system regulating glucocorticoid production and maintaining physiological homeostasis (1).

During pregnancy, however, maternal glucocorticoid regulation is not governed by the HPA axis alone. Instead, the maternal HPA axis interacts with placental CRH signalling to form a dynamic maternal–placental–foetal regulatory network. This pregnancy-specific adaptation involves progressive changes in glucocorticoid physiology that support maternal adaptation, placental function, foetal development, and preparation for parturition (1, 19, 21).

### 3.1 Normal HPA axis adaptation during pregnancy

During pregnancy, the maternal HPA axis undergoes substantial physiological remodelling, with placental corticotropin-releasing hormone (pCRH) serving as a central regulator. Unlike the non-pregnant state, the placenta becomes an important source of CRH, establishing a pregnancy-specific placenta–maternal HPA-axis regulatory network. pCRH stimulates maternal ACTH secretion and contributes to increased cortisol production during gestation (1).

Circulating pCRH concentrations rise progressively from mid-gestation, with a marked increase during late pregnancy and peak levels around delivery (1). Maternal cortisol concentrations similarly increase and may reach approximately two- to fivefold higher levels than in non-pregnant individuals (1). Importantly, this elevation represents a physiological adaptation rather than pathological HPA-axis activation, supporting foetal maturation, placental function, and preparation for parturition (1, 19, 21). Placental glucocorticoid metabolism further regulates foetal exposure to maternal cortisol through mechanisms including 11β-HSD2-mediated glucocorticoid inactivation (1, 12, 17, 20, 22).

Maternal HPA-axis regulation during pregnancy is therefore a dynamic process influenced by gestational timing, placental endocrine signalling, glucocorticoid receptor pathways, placental metabolism, and maternal physiological context (1, 12, 17, 22). Consequently, maternal cortisol concentrations should not be interpreted in isolation as indicators of psychological stress, but rather as components of a broader maternal–placental–foetal regulatory system (1, 4, 17, 20).

### 3.2 Psychological distress and HPA-axis alterations

When interpreting associations between maternal psychological distress and offspring development, psychological exposures, biological markers, and physiological regulatory systems should be distinguished. Perceived stress reflects subjective appraisal of external demands, whereas depression and anxiety represent persistent psychological symptom states. In contrast, cortisol reflects HPA-axis activity but is also influenced by gestational age, circadian regulation, placental function, metabolic status, and measurement methods (4, 23, 24). Therefore, psychological distress, HPA-axis activity, and cortisol levels represent interconnected but distinct biological dimensions rather than interchangeable indicators (1, 4, 23).

Prenatal psychological distress, including stress, depressive symptoms, and anxiety, has been associated with alterations in maternal HPA-axis regulation, including changes in CRH, ACTH, and cortisol profiles (1, 4, 25). However, these associations vary according to exposure characteristics, gestational timing, individual susceptibility, and placental regulatory capacity (1, 17, 22, 23). Importantly, psychological distress may influence HPA-axis sensitivity, feedback regulation, and placental glucocorticoid metabolism rather than simply producing persistent cortisol elevation (1, 4, 17, 22).

Evidence linking maternal psychological distress with foetal glucocorticoid exposure remains inconsistent. Vuppaladhadiam et al. found no significant associations between maternal perceived stress scores and maternal or foetal cortisol/cortisone concentrations or birth weight, suggesting potential placental buffering under healthy pregnancy conditions (20). However, the mechanisms underlying this protection require further investigation using placental glucocorticoid metabolism and receptor-related measures (17, 22).

Other studies have reported altered maternal cortisol profiles in association with stress, anxiety, or depressive symptoms, although findings remain heterogeneous (4, 23, 25). Differences in psychological assessment, biological matrices, sampling timing, and acute versus cumulative glucocorticoid exposure may contribute to these inconsistencies (4, 24, 26).

Overall, prenatal psychological distress should be considered a context-dependent influence on maternal HPA-axis regulation rather than a direct predictor of cortisol elevation (4, 23, 25). Associations between maternal distress, glucocorticoid regulation, and offspring development may additionally be modified by genetic susceptibility, socioeconomic conditions, maternal health behaviours, and postnatal caregiving environments (2, 3, 15). Current human evidence therefore remains insufficient to establish a direct causal pathway from maternal psychological distress through HPA-axis alterations to offspring developmental outcomes (2, 3).

### 3.3 Sex-specific placental adaptation

Increasing evidence suggests that foetal sex modifies the relationship between maternal stress exposure, placental glucocorticoid regulation, and offspring developmental vulnerability (14, 27, 28). The placenta is not a passive barrier but an adaptive interface that may adjust endocrine, immune, and metabolic responses according to foetal sex (14, 27). Such sex-specific adaptations may contribute to differences in developmental trajectories following prenatal environmental exposure (14, 27, 28).

Some studies suggest that foetal sex may influence maternal HPA-axis adaptation during pregnancy. Data from the ABCD cohort indicated that maternal cortisol concentrations differed according to foetal sex, with higher levels observed in pregnancies carrying female foetuses, whereas maternal depressive symptoms alone did not fully explain these differences (23). Another study reported enhanced maternal basal stress responses and cortisol awakening responses in pregnancies with female foetuses (29). However, most studies have not directly tested sex-by-stress interactions, and whether foetal sex modifies the effects of maternal psychological distress on HPA-axis regulation remains unclear (23, 29).

Placental glucocorticoid metabolism represents one potential mechanism underlying sex-specific adaptation. Although 11β-HSD2-mediated conversion of cortisol to cortisone limits foetal glucocorticoid exposure (30, 31), HSD11B2 expression alone does not necessarily indicate functional glucocorticoid barrier capacity. Protein abundance, enzymatic activity, cortisol/cortisone balance, substrate availability, and epigenetic regulation may all contribute to placental glucocorticoid regulation (12, 17, 22). Therefore, sex-specific differences require integrated molecular and functional assessment rather than interpretation based on single biomarkers.

Current evidence suggests that male and female foetuses may differ in vulnerability pathways, with inflammation-related and glucocorticoid-related mechanisms potentially contributing differently to developmental adaptation (32). However, available human studies remain largely observational and do not establish a causal pathway from maternal stress exposure through sex-specific placental glucocorticoid regulation to offspring neurodevelopmental outcomes (17, 22).

Further studies integrating placental transcriptomics, functional glucocorticoid measurements, and longitudinal developmental follow-up are required to clarify how foetal sex shapes maternal–placental–foetal adaptation and developmental susceptibility.

## 4 Assessment of maternal–foetal HPA axis activity

Advances in cortisol measurement technologies have improved assessment of maternal–placental–foetal HPA-axis regulation during the perinatal period (1, 24). However, different biological matrices capture distinct temporal windows and biological dimensions of glucocorticoid exposure and should not be considered interchangeable (24, 26).

Liquid chromatography–tandem mass spectrometry (LC-MS/MS) has improved steroid profiling during pregnancy by enabling sensitive and simultaneous measurement of multiple glucocorticoid-related metabolites (33). In addition, non-invasive biomarkers such as hair cortisol concentration (HCC) and nail cortisol concentration (NCC) provide measures of cumulative glucocorticoid exposure over weeks to months, whereas serum and salivary cortisol primarily reflect short-term fluctuations (24, 26) (**Table 1**).

**Table 1:** Summary of biological samples used for assessing maternal–foetal HPA-axis activity and glucocorticoid exposure during the perinatal period.

Assessment approach	Temporal window	Primary biological information	Biological source	Advantages	Limitations
Salivary cortisol	Minutes to hours	Short-term HPA-axis activity and cortisol dynamics	Maternal free cortisol	Non-invasive; allows repeated sampling; useful for diurnal rhythm and stress reactivity assessment	Strongly influenced by circadian rhythm, sampling timing, acute stress, and compliance
Serum cortisol	Immediate (single time point)	Circulating glucocorticoid concentration	Maternal circulation	Quantitative measurement with high analytical accuracy	Invasive sampling; procedure-related stress may influence levels; reflects only a limited time point
Hair cortisol concentration (HCC)	Weeks to months	Cumulative glucocorticoid exposure	Maternal or offspring hair	Provides retrospective assessment of long-term exposure; minimally invasive	Cannot capture acute changes or cortisol dynamics; affected by hair characteristics and external factors
Nail cortisol concentration (NCC)	Several months	Long-term cumulative cortisol exposure	Maternal or offspring nails	Convenient sampling; potentially reflects prolonged exposure	Limited methodological standardization and fewer validation studies compared with hair cortisol
Placental tissue markers	Pregnancy period/delivery	Placental glucocorticoid regulation and local metabolism	Placental tissue	Enables evaluation of mechanisms including 11β-HSD2 expression/activity and glucocorticoid metabolism	Usually available only at delivery; may not represent earlier pregnancy changes
Cord blood cortisol	At birth	Foetal glucocorticoid environment immediately before birth	Foetal circulation	Provides direct assessment of foetal circulating cortisol exposure near delivery	Influenced by labour, delivery mode, gestational age, and perinatal stress
Neonatal hair cortisol concentration	First months after birth	Early postnatal HPA-axis adaptation and cumulative exposure	Infant hair	Provides retrospective information on early-life glucocorticoid exposure	Limited standardization; interpretation during rapid developmental changes remains challenging

Hair and nail cortisol measurements provide retrospective indicators of cumulative exposure, while offspring hair cortisol studies have suggested associations between prenatal maternal psychological distress and later glucocorticoid profiles in children (24, 26, 34, 35). Although neonatal cortisol declines rapidly after birth during adaptation to the extrauterine environment (36), prenatal glucocorticoid exposure may influence subsequent HPA-axis regulation (30, 37). These findings are consistent with developmental programming hypotheses but do not establish causal programming effects (30, 38).

Importantly, cortisol measures differ substantially in biological interpretation (24). Salivary and serum cortisol reflect short-term HPA-axis dynamics, whereas hair and nail cortisol capture cumulative exposure (24, 26). Placental markers and cord blood cortisol provide additional information regarding foetal exposure and placental glucocorticoid regulation (17, 22). Therefore, no single biomarker can fully represent maternal–placental–foetal HPA-axis activity. Future studies should integrate multiple biological matrices, repeated gestational assessments, placental functional markers, and longitudinal developmental outcomes.

## 5 Maternal HPA-axis regulation and offspring developmental outcomes

Pregnancy involves profound endocrine adaptations in both the mother and foetus (17, 19). As the primary effector hormone of the HPA axis, cortisol contributes to maternal stress responses, placental function, foetal maturation, and neurodevelopment (17, 30). Alterations in maternal HPA-axis regulation have therefore been proposed as one potential pathway linking prenatal environments with offspring developmental variation (17, 39).

However, this relationship should not be interpreted as a simple direct transfer of maternal stress signals to the developing foetal brain (17, 22). Foetal glucocorticoid exposure is shaped by the timing, magnitude, and duration of maternal HPA-axis activity, together with placental glucocorticoid metabolism, foetal susceptibility, and postnatal environmental influences (15, 17, 22, 30). Thus, developmental outcomes likely reflect interactions among endocrine, placental, biological, and environmental pathways rather than a single causal sequence.

Accordingly, this section examines evidence across developmental stages, including (1) foetal and neonatal glucocorticoid exposure and stress-system regulation, (2) infancy and childhood HPA-axis development, and (3) cognitive, emotional, behavioural, and neuropsychiatric outcomes across later development.

The placenta represents a critical interface between the maternal environment and foetal development (17, 20). During pregnancy, it functions not only as an endocrine organ but also as a dynamic regulatory system controlling maternal–foetal glucocorticoid transfer (17, 22). Because the foetal HPA axis remains immature during early and mid-gestation (17, 30), placental regulation of glucocorticoid metabolism, hormone signalling, immune-inflammatory responses, and epigenetic processes is essential for maintaining appropriate foetal glucocorticoid exposure (13, 17).

Seckl and Holmes proposed that excessive or insufficient glucocorticoid exposure during sensitive developmental windows may influence endocrine maturation and contribute to long-term disease susceptibility (30). In this context, placental 11β-HSD2, which converts active cortisol into inactive cortisone, represents an important mechanism limiting maternal glucocorticoid transfer to the foetus (17, 22). However, placental regulation involves multiple interacting processes, including glucocorticoid receptor signalling, local cortisol metabolism, and epigenetic regulation, rather than a single protective pathway (13, 17, 22).

Importantly, the placenta should be viewed as an adaptive organ that responds dynamically to maternal and foetal signals rather than as a passive barrier (17, 20). Alterations in maternal psychological distress, HPA-axis activity, and placental glucocorticoid regulation may interact to influence foetal developmental trajectories (17, 22). Changes in placental glucocorticoid buffering capacity and signalling pathways may modify foetal exposure to glucocorticoids and potentially affect maturation of foetal stress-response systems (13, 17, 22).

However, evidence from human studies remains largely observational (22). Current findings support hypotheses of developmental adaptation and programming, in which prenatal environments may influence developmental trajectories through placental endocrine regulation (17, 30). Nevertheless, available evidence does not establish a definitive causal pathway linking maternal psychological distress, placental glucocorticoid dysregulation, altered foetal exposure, and subsequent neurodevelopmental outcomes (17, 22).

### 5.1 Placental glucocorticoid barrier function: the role of 11β-HSD2

Cortisol is the principal endogenous glucocorticoid and plays essential roles in maternal metabolic regulation, immune adaptation, placental function, and foetal organ maturation (17, 30). However, the developmental effects of glucocorticoids depend not only on exposure levels but also on gestational timing, duration, tissue sensitivity, and placental regulatory capacity (17, 22).

Among the mechanisms regulating placental glucocorticoid metabolism, 11β-HSD2 is one of the most extensively studied pathways (17, 22). By converting biologically active cortisol into inactive cortisone, 11β-HSD2 limits maternal glucocorticoid transfer to the foetus and contributes to maintenance of foetal glucocorticoid homeostasis (12, 17).

Importantly, placental 11β-HSD2 function cannot be inferred solely from HSD11B2 gene expression. Transcriptional regulation, protein abundance, enzymatic activity, and cortisol/cortisone ratios represent distinct biological processes that may not change in parallel (12, 22). Therefore, differences among studies reporting alterations in HSD11B2 expression, protein levels, enzyme activity, or glucocorticoid metabolite concentrations should be interpreted separately rather than considered equivalent indicators of altered glucocorticoid barrier function (12, 22).

Placental glucocorticoid regulation also involves complex interactions between CRH signalling, glucocorticoid receptor pathways, and sex-specific adaptation (17, 19, 27). Emerging evidence suggests that male and female placentas may differ in glucocorticoid-related gene regulation, inflammatory responses, and adaptive capacity, potentially contributing to sex-dependent developmental vulnerability following maternal stress exposure (27, 28).

Recent systematic review evidence indicates that associations between prenatal psychological distress and placental HSD11B2 alterations remain inconsistent, likely reflecting differences in gestational timing, exposure assessment, biological context, and analytical approaches (22). Future studies integrating molecular profiling, functional enzyme measurements, and longitudinal developmental assessment are required to clarify the contribution of placental glucocorticoid metabolism to offspring developmental trajectories (22).

### 5.2 Epigenetic regulation of placental glucocorticoid signalling

In addition to enzymatic regulation, placental glucocorticoid signalling may be influenced by epigenetic mechanisms, including DNA methylation, histone modifications, and non-coding RNA regulation (7, 13, 30). As an environmental sensor and developmental regulator, the placenta may respond to maternal psychological distress, altered HPA-axis activity, inflammatory signals, and metabolic conditions through changes in gene regulation (7, 13, 40, 41).

Recent human studies have investigated epigenetic alterations in glucocorticoid-related pathways. Tartour et al. (22) conducted a systematic review and meta-analysis showing that prenatal psychological distress was associated with modest alterations in placental HSD11B2 expression, although substantial heterogeneity existed across populations, exposure definitions, placental sampling strategies, and analytical methods. Therefore, whether these alterations represent a direct mechanism affecting offspring development remains uncertain; they may instead reflect adaptive responses within the maternal–placental–foetal system (13, 22, 40).

Importantly, placental glucocorticoid regulation is unlikely to be determined by a single molecular pathway. HSD11B2-mediated glucocorticoid metabolism interacts with glucocorticoid receptor signalling (NR3C1), FKBP5-mediated receptor regulation, and epigenetic processes that influence cellular glucocorticoid sensitivity (12, 13, 30). Thus, placental adaptation should be considered as an integrated regulatory network involving hormone metabolism, receptor signalling, epigenetic modification, and immune-inflammatory pathways rather than isolated molecular mechanisms.

A recent study examining different placental regions (13) further demonstrated that maternal HPA-axis activity during early pregnancy was associated with methylation changes in glucocorticoid-related genes, including FKBP5, NR3C1, and HSD11B2, in specific placental compartments. Altered FKBP5 methylation in the decidual layer was associated with shorter gestational duration, suggesting potential links between placental epigenetic regulation and pregnancy adaptation. However, direct associations between maternal depressive symptoms and these methylation changes were not consistently observed, indicating that placental epigenetic responses may reflect integrated biological signals rather than psychological symptoms alone.

Overall, current evidence supports a role for placental epigenetic regulation in maternal–foetal biological communication (7, 13, 30). Nevertheless, most human studies remain associative. Future research integrating DNA methylation, gene expression, protein abundance, enzymatic activity, and longitudinal offspring outcomes is required to determine whether epigenetic alterations represent functional mediators of developmental vulnerability (13, 18).

### 5.3 Sex-specific placental adaptation to maternal stress

The capacity of the placenta to regulate maternal glucocorticoid exposure may be influenced by foetal sex (14, 27, 42). Increasing evidence suggests that male and female placentas differ in glucocorticoid metabolism, immune responses, and adaptive strategies, potentially contributing to sex-specific developmental trajectories following prenatal stress exposure (14, 27, 41, 42).

Animal studies provide mechanistic support for this concept. Wieczorek et al. demonstrated that maternal stress increased corticosterone exposure in female foetuses, whereas male foetuses showed greater placental protection through increased expression of 11β-HSD2 and glucocorticoid-efflux transporters (27). These findings suggest that placental adaptation to maternal stress may involve sex-specific regulatory strategies rather than uniform responses.

Human evidence also supports potential sex-dependent pathways. Freedman et al. (32) reported that maternal depressive symptoms at approximately 16 weeks of gestation were associated with reduced neonatal P50 inhibition, but the underlying biological pathways differed according to foetal sex. In male-foetus pregnancies, inflammatory markers were more strongly associated with outcomes, whereas cortisol-related pathways appeared more prominent in female-foetus pregnancies (32).

However, current evidence does not support the conclusion that either sex is universally more vulnerable. Many human studies have not formally tested sex-by-exposure interactions, and available findings remain largely observational (14, 22, 27, 32). Future studies integrating placental transcriptomics, proteomics, functional glucocorticoid measurements, and longitudinal neurodevelopmental assessment are needed to clarify how foetal sex modifies maternal–placental–foetal adaptation (10, 14, 40).

### 5.4 Summary and future directions

Overall, current evidence supports a model in which maternal psychological distress and altered HPA-axis regulation may influence offspring development through interacting placental mechanisms, including glucocorticoid metabolism, epigenetic regulation, and immune-related pathways (13, 17, 22, 39). However, most human studies remain observational and cannot establish a definitive causal pathway between maternal stress exposure, placental alterations, foetal glucocorticoid exposure, and neurodevelopmental outcomes (10, 17, 39).

Future studies should integrate longitudinal endocrine assessments, placental molecular biomarkers, cord blood glucocorticoid profiles, neonatal neuroimaging, electrophysiological measures, and long-term developmental follow-up to better link prenatal biological processes with early brain development (10, 24, 39).

The concept of “foetal programming” should therefore be interpreted cautiously as a framework describing how early environmental conditions may influence developmental trajectories rather than as a fully established causal mechanism in humans (10, 18). Integrated multi-dimensional approaches will be required to distinguish developmental adaptation from persistent biological alterations associated with later vulnerability (10, 13, 39).

### 5.5 Developmental trajectories of offspring HPA-axis regulation

The foetal and offspring HPA axis undergoes progressive maturation from pregnancy through childhood, shaped by the intrauterine endocrine environment, placental regulation, birth transition, and early postnatal experiences (16, 18, 24, 36). Because HPA-axis development is highly stage-dependent, maternal glucocorticoid signalling may have different biological consequences according to the timing, duration, and developmental window of exposure (18, 24).

During pregnancy, the placenta regulates maternal–foetal glucocorticoid communication through hormone metabolism and signalling pathways, while the foetal HPA axis gradually develops under this endocrine environment (16, 24, 30). After birth, withdrawal of placental endocrine influences initiates rapid adaptation of neonatal HPA-axis function, including the establishment of postnatal regulatory rhythms (36, 43). During infancy and childhood, maturation of cortisol rhythms, feedback regulation, and stress responsiveness continues, with potential implications for emotional regulation, behavioural development, and later psychiatric vulnerability (15, 36, 44, 45). Therefore, associations between maternal HPA-axis regulation and offspring outcomes should be interpreted within a developmental-stage-specific framework rather than as a single uniform mechanism across the lifespan (18, 24).

Current human evidence remains largely observational and does not establish a continuous causal pathway linking maternal stress or cortisol alterations, placental regulatory changes, foetal HPA-axis adaptation, and later neuropsychiatric outcomes. Accordingly, existing findings are more appropriately interpreted as consistent with developmental adaptation or programming hypotheses rather than definitive evidence of causal programming in humans (10, 46).

#### 5.5.1 Pregnancy: maternal cortisol signalling, placental buffering, and foetal HPA-axis maturation

During pregnancy, foetal HPA-axis development occurs within a dynamic maternal–placental–foetal endocrine environment. Maternal cortisol progressively increases across gestation, partly driven by rising placental CRH secretion. Although glucocorticoids are essential for foetal maturation, excessive, prolonged, or mistimed exposure may influence the development of foetal stress-regulatory systems. The placenta regulates foetal glucocorticoid exposure through mechanisms including 11β-HSD2-mediated conversion of active cortisol to inactive cortisone, thereby contributing to foetal glucocorticoid homeostasis (16, 18, 24, 30).

Human studies have reported associations between prenatal psychological distress and alterations in placental glucocorticoid regulation; however, findings remain heterogeneous and do not establish a direct causal pathway linking maternal psychological distress with foetal HPA-axis programming (13, 17, 22). Associations between prenatal depressive or anxiety symptoms and maternal cortisol regulation vary according to symptom characteristics, gestational timing, and cortisol assessment methods (4, 23, 25).

Hair cortisol concentration (HCC) provides an indicator of cumulative maternal glucocorticoid exposure but does not directly represent foetal glucocorticoid exposure. Nyström-Hansen et al. reported higher third-trimester HCC among women with severe mental disorders, with poorer current functioning associated with greater cumulative cortisol exposure (47). These findings highlight the value of long-term glucocorticoid biomarkers while emphasising the need to distinguish maternal systemic exposure from foetal endocrine status (24, 47).

Emerging studies have examined associations between maternal psychological distress and early offspring glucocorticoid profiles. Karl et al. reported that higher maternal prenatal depressive symptoms were associated with higher neonatal hair cortisol concentrations measured shortly after birth, although associations with cortisol/cortisone ratios were not significant. Whether these early hormonal patterns represent persistent alterations in HPA-axis regulation or predict later developmental outcomes requires longitudinal investigation (48).

Similarly, Van Zundert et al. reported associations between higher maternal first-trimester hair cortisol concentrations and reduced embryonic growth trajectories; however, this study did not directly assess foetal HPA-axis maturation or establish causality (49).

Overall, current evidence suggests that maternal psychological distress and cortisol-related measures may be associated with variation in the early developmental endocrine environment. However, whether maternal cortisol exposure, placental regulation, or neonatal glucocorticoid profiles represent adaptive responses, biomarkers of vulnerability, or causal mediators remains unresolved (17, 48, 49).

#### 5.5.2 Birth and neonatal period: biological evidence of intrauterine glucocorticoid exposure

Direct assessment of foetal HPA-axis activity is technically challenging in humans; therefore, biomarkers collected at or shortly after birth provide indirect information about the intrauterine glucocorticoid environment. These include cord blood cortisol, neonatal hair cortisol, and neonatal nail cortisol. However, each biological matrix reflects different temporal windows and biological dimensions of glucocorticoid exposure and should not be considered interchangeable (24).

The birth process itself may substantially influence neonatal glucocorticoid measurements. Cord-blood cortisol is often higher following vaginal delivery than elective caesarean delivery, although findings are not entirely consistent. Labour and delivery conditions may also affect placental glucocorticoid metabolism. Therefore, obstetric factors, gestational age, and sampling conditions should be carefully considered when interpreting neonatal endocrine biomarkers (12, 50).

Karl et al. reported that higher maternal prenatal depressive symptoms were associated with increased neonatal hair cortisol concentrations, whereas no significant associations were observed for hair cortisone and only a non-significant trend for the cortisol/cortisone ratio. These findings suggest that maternal prenatal psychological symptoms may be associated with altered early-life glucocorticoid profiles; however, the underlying placental and foetal mechanisms remain unclear, and the predictive value of these early hormonal signatures requires longitudinal investigation (48).

In contrast, Vuppaladhadiam et al. (20) found no significant associations between maternal perceived stress and maternal or foetal cortisol or cortisone concentrations. These findings are compatible with the possibility that placental or other maternal–foetal regulatory mechanisms buffer foetal steroid exposure from variations in perceived maternal stress, although the specific molecular mechanisms were not directly assessed.

Overall, neonatal glucocorticoid biomarkers provide complementary information regarding the late-gestational and perinatal endocrine environment. Their interpretation requires consideration of placental regulation, gestational age, labour and delivery conditions, maternal medication exposure, obstetric complications, sampling timing, and biological matrix. For measurements obtained later in infancy, additional postnatal influences, including feeding, sleep, illness, caregiving, and socioeconomic environment, should also be considered (24, 48).

#### 5.5.3 Infancy: maturation of HPA-axis function and early stress responses

Following birth, the infant HPA axis continues to mature and adapt. Although the prenatal endocrine environment may influence early stress-response development, these effects are not fixed and may be modified by postnatal factors, including mother–infant interactions, maternal mental health, feeding, sleep, and caregiving environment. Therefore, prenatal exposures should be considered potential contributors to susceptibility rather than deterministic predictors of later HPA-axis function (15, 44, 45).

Karl et al. (48) measured cortisol and cortisone concentrations in infant hair and reported that greater maternal depressive symptoms during pregnancy were associated with higher neonatal hair cortisol concentrations. However, this study did not directly assess foetal glucocorticoid exposure or infant HPA-axis function, and the long-term significance of these early hormonal differences remains unclear.

Downes et al. (34) investigated longitudinal associations between maternal depressive and anxiety symptoms and child hair glucocorticoids in the EDEN birth cohort. Prenatal depressive symptoms were associated with slightly higher hair cortisone at 1 year, whereas prenatal anxiety symptoms were associated with higher hair cortisol at birth and higher hair cortisone at birth and 1 year. These findings suggest that psychological exposure type, developmental timing, and biomarker selection may influence observed associations; however, they do not establish persistent HPA-axis dysregulation.

Evidence from later developmental stages has also been inconsistent. Cohen et al. (51), using data from the Project Viva and Generation R cohorts, found no significant associations between high prenatal depressive symptoms and cord-blood glucocorticoids or child hair cortisol from childhood to early adolescence. Together with findings from Karl et al. (48), Downes et al. (34), and Chortatos et al. (52), these results indicate that associations vary according to exposure characteristics, biological matrix, developmental stage, and analytical approaches.

Overall, current evidence is more consistent with a developmental adaptation framework than with a deterministic interpretation of prenatal programming. Maternal psychological and endocrine conditions may contribute to variation in offspring glucocorticoid regulation, but these associations are time-dependent, method-sensitive, and influenced by postnatal environments. Future studies integrating placental biomarkers, repeated endocrine assessments, postnatal environmental measures, and long-term neurodevelopmental follow-up are needed to clarify HPA-axis developmental trajectories (15, 34, 48, 51).

#### 5.5.4 Childhood: HPA-axis regulation and neuropsychiatric developmental outcomes

During childhood, HPA-axis regulation continues to mature within the context of caregiving, social environment, and broader developmental experiences, contributing to individual differences in stress reactivity and emotional–behavioural functioning (15, 44). Beyond HPA-axis biomarkers, longitudinal studies have extensively examined associations between maternal perinatal psychological distress and offspring developmental outcomes. Rogers et al. (3) synthesised prospective cohort studies and reported associations between maternal perinatal depression and anxiety and poorer offspring outcomes across social-emotional, cognitive, language, motor, and adaptive-behaviour domains. However, the magnitude and consistency of these associations varied across developmental domains and exposure periods, and available evidence does not demonstrate that these effects are mediated specifically through HPA-axis dysregulation (3).

From an endocrine biomarker perspective, Plank et al. (53) investigated offspring hair cortisol concentrations during primary-school age and early adolescence. Biomarker-indicated prenatal alcohol exposure was associated with lower childhood hair cortisol concentrations, although this difference was attenuated during adolescence. In contrast, maternal prenatal depressive symptoms were not significantly associated with offspring hair cortisol at either developmental stage. These findings suggest that later-life glucocorticoid profiles may vary according to exposure type, developmental timing, and biomarker assessment methods (53).

Overall, maternal perinatal psychological distress has been associated with diverse neurodevelopmental outcomes, whereas evidence for persistent alterations in offspring HPA-axis biomarkers remains limited and inconsistent. Current findings support a developmental adaptation framework in which prenatal conditions may contribute to individual variability in stress-system regulation and neurodevelopment, rather than a deterministic pathway from maternal distress to offspring HPA-axis dysfunction (3, 53).

### 5.6 Neurodevelopmental trajectories and psychiatric vulnerability

Current evidence suggests that prenatal maternal environmental conditions may be associated with variation in offspring stress-system regulation, neurocognitive development, emotional functioning, and psychiatric vulnerability. However, human studies have not established whether alterations in offspring HPA-axis regulation represent a causal mediator linking maternal HPA-axis-related exposures with later developmental outcomes. Rather than indicating a simple or persistent “foetal programming effect”, these associations may reflect dynamic developmental adaptation shaped by maternal endocrine regulation, placental function, foetal developmental stage, and postnatal environmental influences. Therefore, interpretation of maternal HPA-axis-related effects on offspring neuropsychiatric outcomes requires consideration of multiple developmental windows and interacting biological and environmental pathways (2, 10, 39, 46).

#### 5.6.1 Cognitive, language, and motor development

Early neurodevelopment is commonly assessed using standardised instruments such as the Bayley Scales of Infant and Toddler Development, including cognitive, language, and motor domains. Birth-cohort studies have investigated associations among maternal psychological distress, glucocorticoid-related biomarkers, and early developmental outcomes. However, findings remain heterogeneous because of differences in exposure timing, biological measurements, developmental assessments, offspring sex, and adjustment for postnatal environmental factors (3, 11, 46).

Mariño-Narváez et al. (54) examined associations between maternal HCC measured during pregnancy and postpartum and neurodevelopment at 12 months in 93 mother–infant dyads. Maternal HCC was associated with selected cognitive, language, and motor outcomes, with differing patterns between boys and girls. These findings suggest that maternal cortisol exposure within physiological ranges is not uniformly associated with adverse development; however, they do not demonstrate that cortisol directly promotes foetal brain maturation or causes developmental differences (54).

Chan et al. (55) investigated maternal perceived-stress and hair cortisol trajectories from preconception to late pregnancy in relation to neonatal nucleus accumbens microstructure. Higher perceived-stress trajectories, particularly before conception, were associated with differences in neonatal nucleus accumbens orientation dispersion index, whereas maternal hair-cortisol trajectories were not associated with corresponding microstructural differences. Although neonatal brain measures moderated associations with later externalising behaviour at 3–4 years, these findings do not establish a cortisol-mediated developmental pathway (55).

Neonatal P50 inhibition provides an early neurophysiological indicator of developing inhibitory function. Freedman et al. (32) reported associations between maternal depressive symptoms and newborn P50 inhibition, with inflammation-related associations more evident in male-foetus pregnancies and cortisol-related associations more evident in female-foetus pregnancies. Although early gestation represents a period of active neurodevelopment, these findings do not establish a specific causal window through which maternal biological signals determine later neurophysiological function (32, 56, 57).

Sex-specific developmental patterns have also been reported. Freedman et al. (32) suggested different inflammation- and cortisol-related pathways according to foetal sex, while experimental placental studies have demonstrated sex-dependent glucocorticoid responses (27, 32, 42). However, current evidence does not support the conclusion that male offspring are generally inflammation-sensitive or female offspring universally glucocorticoid-sensitive.

Mumm et al. (58) examined maternal third-trimester serum cortisol and early language development in 1,093 mother–child dyads. Higher maternal cortisol was associated with more advanced productive vocabulary in boys during earlier assessment and better receptive vocabulary in girls at 12–21 months. These findings suggest sex- and domain-specific associations but do not directly demonstrate effects on brain maturation (58).

Chen et al. (59) reported that persistent moderate or high maternal depressive and anxiety symptom trajectories were associated with increased risk of delays in communication, motor, problem-solving, and personal–social domains during the first 24 months. However, because the study assessed psychological symptoms rather than cortisol or placental glucocorticoid regulation, these findings cannot be interpreted as evidence for an HPA-axis-mediated mechanism (59).

Overall, maternal psychological distress and glucocorticoid-related biomarkers have been associated with cognitive, language, motor, neurophysiological, and brain-development outcomes, but associations vary according to exposure timing, measurement methods, developmental domains, and offspring sex. Physiological glucocorticoid signalling is essential for development, and higher cortisol concentrations within observed human ranges are not consistently associated with adverse outcomes. Current evidence does not establish maternal cortisol as a direct mediator between psychological distress and offspring neurodevelopment. Future studies integrating repeated endocrine assessment, placental functional measures, neuroimaging, developmental testing, and postnatal environmental factors are required (11, 54, 55, 58–60).

#### 5.6.2 Emotional and behavioural outcomes

Maternal depressive symptoms during pregnancy have been associated with offspring emotional and behavioural outcomes; however, the biological pathways underlying these associations remain unclear. Foetal heart-rate variability (FHRV), reflecting aspects of developing autonomic regulation, has been investigated as an early marker of foetal physiological adaptation. Although maternal psychological state and glucocorticoid regulation may influence FHRV, current evidence does not establish a direct pathway from maternal depression through cortisol exposure to later behavioural outcomes (3, 61).

In a cross-sectional study of primiparous women during the third trimester, Turan and Kaya (62) reported a positive association between maternal salivary cortisol and foetal heart rate. However, the study assessed foetal heart rate rather than FHRV, and its design did not allow causal interpretation. Hunter et al. (61) investigated 23 pregnancies at 28–33 weeks and found that greater maternal depressive symptoms were associated with lower FHRV, particularly in male foetuses. Lower cortisol-to-total-corticosteroid ratios were also associated with reduced FHRV, which was subsequently related to poorer newborn sensory gating and infant self-regulation. These findings suggest potential links among maternal mood, corticosteroid balance, and foetal autonomic development, although the small sample size limits interpretation (61).

Mariño-Narváez et al. examined associations among maternal psychological risk, HCC, and child outcomes in 56 mother–child dyads. Although high-risk pregnancies were characterised by greater perceived stress and pregnancy-specific distress, maternal pregnancy HCC did not differ significantly between groups. At 24 months, behavioural differences were observed, including higher internalising scores in children from the high-risk group; however, prenatal cortisol was not demonstrated to mediate these outcomes (54, 63).

Mustonen et al. (64) examined maternal HCC during mid- and late pregnancy in relation to child socioemotional problems at 2 and 5 years. Higher late-pregnancy HCC was associated with fewer total socioemotional problems at 2 years, whereas associations were not significant at 5 years. Additional associations with internalising and emotional regulation outcomes were modest and mainly identified in stratified or interaction analyses. These findings indicate that higher maternal cortisol within observed physiological ranges is not uniformly associated with adverse socioemotional development (64).

Byg et al. (65) assessed maternal third-trimester serum cortisol and preschool internalising problems. Although positive associations were observed in unadjusted analyses, they were no longer significant after adjustment for parental psychiatric history, demographic factors, smoking, parity, gestational age, and other covariates. These findings do not support maternal cortisol as an independent predictor of preschool emotional difficulties (65).

Overall, current evidence does not support a simple linear model in which maternal psychological distress leads to increased cortisol exposure and subsequently causes adverse emotional or behavioural outcomes. Associations vary according to cortisol measurement method, gestational timing, offspring age and sex, outcome domain, and prenatal or postnatal environmental factors. Maternal cortisol-related findings should therefore be interpreted within a broader developmental framework involving placental regulation, immune and metabolic conditions, genetic susceptibility, and caregiving environments. Physiological glucocorticoid signalling is essential for foetal maturation, and current observational studies cannot determine whether altered cortisol patterns represent adaptive responses, markers of maternal–foetal conditions, or causal contributors to behavioural variation. Future research should integrate repeated endocrine assessments, placental functional markers, foetal neurophysiological measures, and longitudinal behavioural follow-up (10, 18, 37, 38, 61, 64, 65).

#### 5.6.3 Psychiatric disorders

Increasing epidemiological evidence suggests that maternal psychological stress, anxiety, and depressive symptoms during pregnancy (66) are associated with a range of offspring emotional, behavioural, and psychiatric outcomes. However, associations with specific neurodevelopmental diagnoses remain heterogeneous, and maternal cortisol has not been established as a direct mediator. Any contribution of maternal glucocorticoid signalling is likely to operate within a broader network involving placental glucocorticoid metabolism, genetic susceptibility, immune-inflammatory processes, treatment exposure, and postnatal environmental conditions (2, 3, 67, 68).

Population-based studies by Tusa et al. (68) reported that maternal perinatal depressive disorders were associated with increased risks of depressive and anxiety disorders, as well as ADHD, in offspring. These studies did not establish cortisol or offspring HPA-axis dysregulation as mediators; the reported associations may also reflect familial genetic liability, maternal illness severity, treatment exposure, and shared postnatal environment (68).

Maternal steroid hormone profiles, including cortisol, have also been investigated in relation to neurodevelopmental disorders such as ASD; however, current evidence is limited and does not support a simple relationship in which greater prenatal cortisol exposure uniformly increases risk (69). ASD has a substantial genetic component and marked phenotypic heterogeneity (70, 71). Maternal hormonal associations should therefore be interpreted as potential contextual contributors rather than as alternatives to genetic liability.

Kosidou et al. (69) examined first-trimester maternal oestradiol, testosterone, 17-hydroxyprogesterone, and cortisol in relation to diagnosed autism with and without intellectual disability. Lower oestradiol and higher cortisol were respectively associated with higher and lower odds of autism with intellectual disability, whereas higher 17-hydroxyprogesterone was associated with greater odds of autism without intellectual disability. The associations were nonlinear and differed according to co-occurring intellectual disability. The study found no evidence that associations differed by offspring sex and did not directly assess genetic susceptibility. These findings indicate that early maternal steroid profiles may be associated with specific autism phenotypes, but they do not support a simple model in which higher maternal cortisol increases overall ASD risk.

Overall, maternal psychological disorders and steroid-hormone profiles have been associated with several offspring psychiatric and neurodevelopmental outcomes, but the direction and specificity of findings vary across diagnoses and biological markers. Current evidence does not establish maternal cortisol as a direct mediator of ASD, ADHD, anxiety, or depressive disorders. Genetic liability, phenotypic heterogeneity, placental and immune regulation, treatment exposure, and the postnatal family environment must be considered alongside prenatal endocrine measures. Longitudinal studies integrating maternal endocrine profiles, genetic and epigenetic susceptibility, placental biology, and repeated psychiatric assessment are required to clarify the contribution of maternal HPA-axis regulation (2, 68–70).

#### 5.6.4 Developmental timing of maternal HPA-axis regulation and offspring outcomes

From pregnancy through early childhood, maternal HPA-axis regulation, placental glucocorticoid metabolism, and offspring stress-response systems undergo continuous developmental change. Therefore, the biological significance of maternal psychological distress or glucocorticoid exposure is likely to depend on exposure timing, duration, and developmental context. Similar maternal stress or cortisol measures obtained at different stages may consequently show different, and sometimes opposing, associations with offspring outcomes. However, current human evidence does not demonstrate that exposure timing alone explains these differences, as gestational stage is closely linked to changes in placental function, foetal maturation, maternal physiology, and measurement approaches (10, 11, 18).

During pregnancy, maternal cortisol secretion, placental CRH signalling, and 11β-HSD2-mediated glucocorticoid metabolism jointly contribute to the maternal–placental–foetal endocrine environment, with their relative contributions changing across gestation. Importantly, glucocorticoid biomarkers represent different biological dimensions rather than equivalent measures of a single exposure. Maternal serum or salivary cortisol reflects short-term circulating levels, whereas hair cortisol provides an estimate of cumulative exposure. Cord-blood cortisol reflects the perinatal endocrine environment and is influenced by labour and delivery conditions, while neonatal hair glucocorticoids may integrate late-gestational and early postnatal exposure. Later childhood cortisol measures reflect offspring HPA-axis regulation within a substantially different developmental and environmental context. These biomarkers should therefore not be interpreted as sequential indicators of one continuous causal pathway (12, 16, 24, 30, 48, 51).

Offspring HPA-axis regulation and developmental outcomes also vary across developmental stages. During foetal and neonatal life, associations may involve glucocorticoid exposure, autonomic regulation, and early physiological adaptation. During infancy, maturation of circadian rhythms, cortisol reactivity, and recovery patterns provide additional indicators of stress-system development. In childhood, associations may emerge in later cortisol regulation, cognitive and language development, emotional–behavioural functioning, or psychiatric symptoms. These developmental differences likely reflect both maturation of the offspring HPA axis and increasing influences from postnatal caregiving, sleep, feeding, socioeconomic conditions, and other environmental factors. Therefore, maternal HPA-axis-related measures and offspring outcomes should be interpreted within developmental-stage-specific frameworks rather than as evidence of a single deterministic causal process (3, 34, 36, 45, 48, 58).

**Table 2** summarises representative maternal–placental–offspring HPA-axis-related indicators, corresponding biological processes, developmental outcomes, and key interpretive considerations across prenatal and postnatal developmental stages.

**Table 2:** Developmental timing of maternal–placental–offspring HPA-axis-related indicators, biological processes, developmental outcomes, and interpretive considerations.

Developmental stage	Representative indicators	Principal biological context	Representative outcomes or assessments	Main interpretive considerations
Early pregnancy	Maternal serum, salivary, or hair cortisol; maternal psychological and HPA-axis measures; early placental molecular markers	Placental establishment, early embryonic growth, and early placental endocrine or epigenetic adaptation	Embryonic growth trajectories, placental molecular variation, and gestational development	Evidence is predominantly observational; early placental tissue is rarely available, and exposure timing is difficult to isolate
Mid-pregnancy	Maternal cortisol trajectories; increasing pCRH; placental HSD11B2/11β-HSD2-related measures	Placental glucocorticoid regulation and developing foetal HPA, autonomic, and neural systems	Foetal autonomic or neurophysiological indicators and subsequent developmental outcomes	Mechanistic evidence is stronger in experimental models; human placental markers are commonly measured only at delivery
Late pregnancy	Maternal cortisol rhythm and trajectory; serum, salivary, or hair cortisol; pCRH; foetal heart rate and FHRV	Foetal organ maturation, preparation for parturition, and late-gestational endocrine adaptation	Foetal neurophysiological measures, birth outcomes, and neonatal glucocorticoid profiles	Physiological increases in cortisol and pCRH complicate interpretation; findings are sensitive to sampling time and maternal or obstetric factors
Birth and neonatal period	Cord-blood cortisol and cortisone; placental glucocorticoid markers; neonatal hair cortisol and cortisone	Perinatal endocrine transition, placental metabolism at delivery, and early extrauterine adaptation	Neonatal glucocorticoid profiles, birth adaptation, and early neurophysiological measures	Strongly influenced by labour, delivery mode, gestational age, sampling conditions, and the exposure window represented by neonatal hair
Infancy	Infant hair or salivary cortisol; diurnal rhythm; cortisol reactivity and recovery	Emerging HPA-axis rhythms and feedback regulation, with continued postnatal plasticity	Early stress regulation, socioemotional functioning, and behavioural adaptation	Maternal mental health, caregiving, feeding, sleep, illness, and developmental age may modify observed associations
Childhood	Offspring hair or salivary cortisol; diurnal rhythm; stress reactivity and recovery	Later HPA-axis regulation within increasingly complex social and environmental contexts	Cognitive and language development, emotional and behavioural functioning, and psychiatric symptoms	Findings are exposure- and age-specific, often inconsistent, and vulnerable to genetic, socioeconomic, family, and postnatal confounding

As illustrated in **Table 2**, associations between HPA-axis-related measures and offspring outcomes vary according to the timing of exposure, biological sampling, and developmental assessment.

These stage-specific differences are reflected across the literature. Early pregnancy studies have mainly examined maternal endocrine conditions, placental molecular regulation, and early embryonic development, whereas mid-to-late pregnancy studies have focused more on maternal cortisol dynamics, placental glucocorticoid buffering, foetal autonomic and HPA-axis maturation, and neurodevelopmental processes. Around birth and during infancy, prenatal endocrine conditions may be reflected in neonatal glucocorticoid biomarkers, neurophysiological responses, and early stress regulation, while later infancy and childhood studies have examined cortisol regulation, cognitive and language development, and emotional–behavioural outcomes. However, these findings are derived from different cohorts, exposure definitions, biological matrices, and developmental assessments and should not be interpreted as a single continuous pathway from placental alterations to foetal HPA-axis remodelling and later neuropsychiatric disorders (13, 48, 49, 55, 58, 61).

Current human evidence remains largely observational and heterogeneous regarding exposure definitions, sampling strategies, glucocorticoid assays, confounder adjustment, and follow-up duration. Many studies assess only a single biomarker at one developmental stage, limiting differentiation between transient adaptation and persistent developmental alteration. Future research should integrate longitudinal endocrine profiling, placental molecular and functional assessment, neonatal biomarkers, offspring stress reactivity, neuroimaging or electrophysiological measures, and postnatal environmental factors. Such approaches may clarify sensitive developmental windows and determine whether observed associations represent causal mechanisms, adaptive responses, or correlated biomarkers (10, 39, 46).

## 6 Mechanisms beyond cortisol

Beyond glucocorticoid signalling, associations between maternal psychological distress and offspring developmental outcomes are likely to involve multiple interacting biological pathways (2, 18, 39). Maternal HPA-axis activity and cortisol regulation represent important components of prenatal adaptation; however, cortisol concentrations should not be interpreted as a uniform biological signature of stress exposure or as the sole mediator linking maternal psychological states with offspring development (2, 4, 10). Instead, developmental outcomes are likely shaped by interactions among endocrine, immune-inflammatory, metabolic, and epigenetic processes within the maternal–placental–foetal system (7, 40).

The placenta represents a central regulatory interface within this network, integrating glucocorticoid metabolism with immune signalling, nutrient transport, oxidative balance, and epigenetic regulation (7, 40, 72). Under physiological conditions, these regulatory mechanisms may buffer fluctuations in maternal psychological state and maintain foetal homeostasis. For example, some studies of healthy pregnancies have not identified significant associations between maternal perceived stress and maternal or foetal cortisol/cortisone concentrations, suggesting that maternal–placental–foetal regulatory processes may attenuate variation in psychological stress exposure. However, such findings do not directly demonstrate active compensatory mechanisms, and the extent to which placental buffering capacity is altered under conditions of chronic stress, inflammation, metabolic disturbance, or placental dysfunction remains unclear (20, 40).

Therefore, observed associations between maternal stress-related exposures and offspring developmental outcomes should be interpreted within a dynamic multisystem framework rather than as consequences of a single cortisol-mediated pathway. Subsequent sections discuss additional mechanisms, including epigenetic regulation, immune-inflammatory pathways, metabolic factors, and other biological processes that may contribute to developmental vulnerability and adaptation.

### 6.1 Epigenetic regulation of placental glucocorticoid signalling

Beyond variation in glucocorticoid-metabolising enzyme activity, placental glucocorticoid signalling may also be regulated through epigenetic processes. DNA methylation has been directly investigated in human placental glucocorticoid-related genes, whereas evidence concerning histone modifications and non-coding RNA regulation is derived more broadly from placental and developmental epigenetic research. These processes may influence the regulation of genes involved in glucocorticoid metabolism, receptor sensitivity, and intracellular signalling, although their functional consequences in human placental adaptation remain incompletely understood. However, most human evidence remains observational, and epigenetic differences should currently be interpreted as molecular correlates of maternal endocrine and environmental conditions rather than as confirmed adaptive responses or causal programming mechanisms (7, 13, 30).

Castro-Quintas et al. (13) investigated DNA methylation of glucocorticoid-related genes (FKBP5, NR3C1, and HSD11B2) in chorionic villous and maternal decidual placental compartments from 45 mother–infant dyads. Maternal diurnal salivary cortisol and depressive symptoms were assessed during pregnancy. Higher first-trimester maternal cortisol was associated with region-specific methylation differences in NR3C1 and FKBP5, particularly in chorionic villous tissue, whereas depressive symptoms were not significantly associated with methylation of the analysed regions. Decidual FKBP5 methylation was additionally associated with gestational duration (13).

Because placental samples were collected at delivery, the study could not determine when methylation differences emerged or whether early pregnancy cortisol directly induced these changes. The observed associations may reflect sustained maternal endocrine characteristics, cumulative gestational influences, placental adaptation, or unmeasured maternal and obstetric factors. Furthermore, the absence of significant associations between depressive symptoms and methylation does not indicate that psychological distress lacks biological relevance; rather, it reinforces that maternal psychological symptoms and cortisol represent distinct exposure dimensions. Therefore, these findings should be interpreted as associations between maternal endocrine conditions and placental epigenetic patterns rather than evidence of a direct causal epigenetic programming mechanism (13).

FKBP5 encodes a glucocorticoid-receptor co-chaperone that negatively regulates GR signalling and contributes to cellular glucocorticoid sensitivity. However, Castro-Quintas et al. did not measure FKBP5 expression, protein abundance, GR activity, or functional glucocorticoid signalling. Therefore, whether the observed methylation differences altered placental glucocorticoid responsiveness remains unknown. Nevertheless, because the maternal decidua forms part of the maternal–placental interface and participates in pregnancy maintenance and parturition, the region-specific association identifies a biologically plausible target for future functional investigation rather than a confirmed mechanism of preterm birth (13).

Overall, placental epigenetic regulation represents a plausible molecular level at which maternal endocrine conditions may be associated with placental glucocorticoid-related signalling. However, available human studies do not yet establish that placental DNA methylation mediates the relationship between maternal HPA-axis activity and foetal or offspring development. Future studies should integrate DNA methylation with gene expression, protein abundance, receptor function, enzymatic activity, spatially resolved placental analyses, and longitudinal offspring outcomes (7, 13).

### 6.2 Immune-inflammatory and neurotrophic pathways within the maternal–placental–foetal network

Placental glucocorticoid metabolism—particularly 11β-HSD2-mediated cortisol inactivation—is one of the most extensively studied components of maternal–foetal glucocorticoid regulation (12, 22, 30). Beyond placental glucocorticoid metabolism, associations between maternal stress-related exposures and offspring development may involve interacting immune-inflammatory and neurotrophic processes within the maternal–placental–foetal system (2, 72, 73).

Shelton et al. (74) examined 105 pregnant women between 16 and 26 weeks of gestation and reported associations between depressive symptoms, cortisol concentrations, and inflammatory cytokines, although the direction of these associations differed across biomarkers. Other cohorts have reported associations between depressive symptoms and inflammatory markers, including IL-1β, CRP, TNF-α, and IL-6, but findings have been inconsistent across populations and inflammatory measures (75, 76). Collectively, these studies suggest covariance among maternal psychological, endocrine, and immune profiles rather than evidence of a specific placental inflammatory mechanism.

Scott et al. (41) used pregnancies complicated by maternal asthma to examine associations among asthma severity, placental cytokine expression, foetal cortisol, glucocorticoid treatment, and foetal sex. Placental expression of several cytokines was elevated in female-foetus pregnancies complicated by mild asthma, and placental TNF-α, IL-1β, and IL-6 expression was negatively correlated with female cord-blood cortisol. Comparable associations were not observed in male-foetus pregnancies. Importantly, cytokine expression was not significantly associated with inhaled glucocorticoid use. These findings should therefore be interpreted within the context of maternal disease-related inflammation rather than as evidence of adverse effects of therapeutic glucocorticoid exposure. Rather, they demonstrate sex-specific covariance among maternal asthma, placental inflammatory expression, and foetal cortisol (41).

Freedman et al. (32) reported sex-dependent associations among maternal depressive symptoms, inflammatory and glucocorticoid-related biomarkers, and neonatal P50 inhibition. In pregnancies with male foetuses, maternal depression was more closely associated with CRP-related patterns, and CRP was associated with poorer newborn P50 inhibition. In pregnancies with female foetuses, corresponding associations were more evident for maternal cortisol. Cortisol-to-cortisone relationships were also more pronounced in male-foetus pregnancies, suggesting sex-related differences in glucocorticoid metabolism (32). One possible explanation is that male- and female-foetus pregnancies may differ in the relative contribution of inflammatory and glucocorticoid-related signalling pathways. However, this hypothesis remains speculativewhile remaining more closely associated with inflammatory signalling, whereas female-foetus pregnancies may show greater sensitivity to maternal cortisol-related variation. However, this interpretation remains provisional: Freedman et al. (32) did not directly measure placental HSD11B2 expression, 11β-HSD2 enzymatic activity, glucocorticoid transporters, or placental cytokine expression. The study therefore supports sex-dependent biomarker pathways but does not identify the placental mechanism responsible for them (27, 32, 41).

Lamadé et al. (73) examined cord-blood BDNF at birth and found higher foetal BDNF concentrations in association with maternal depressive symptoms and lower socioeconomic status. However, BDNF was not consistently associated with newborn anthropometric outcomes after sensitivity analyses, and the study did not include longitudinal neurodevelopmental follow-up. BDNF is biologically involved in neuronal survival, synaptic development, and neural-network maturation. Cord-blood BDNF should therefore be interpreted as a potential biomarker reflecting neurotrophic signalling at birth rather than evidence of a mechanistic mediator linking maternal stress exposure to later brain development.

Overall, maternal psychological, endocrine, inflammatory, and neurotrophic measures appear to covary during pregnancy, with some associations modified by foetal sex. However, current evidence does not establish that glucocorticoid metabolism, inflammatory cytokines, or BDNF constitute mediating mechanisms underlying later offspring developmental outcomes. These processes should currently be considered interacting candidate pathways and biological correlates within the maternal–placental–foetal system rather than components of a demonstrated causal sequence (32, 41, 73, 74). Future studies integrating repeated maternal endocrine and inflammatory assessments, placental functional measurements, neurotrophic markers, formal sex-by-exposure analyses, and longitudinal offspring follow-up are needed (2, 10).

### 6.3 Maternal–foetal cellular communication and immune-mediated developmental regulation

Bidirectional cellular trafficking occurs during pregnancy, resulting in both foetal microchimerism, referring to the persistence of foetal cells in maternal tissues, and maternal microchimerism, referring to the persistence of maternal cells in offspring tissues. Bianchi et al. (77) provided evidence for the long-term persistence of foetal male cells in maternal blood, whereas broader evidence regarding bidirectional maternal–foetal cellular exchange and its immunological implications has been reviewed by Kinder et al. (78).

Experimental evidence suggests that maternal microchimeric cells may have functional roles in offspring development. In mice, Schepanski et al. (79) demonstrated that maternal microchimeric cells entered the developing offspring brain, contributed to microglial homeostasis, reduced excessive presynaptic elimination, and supported maturation of prefrontal–hippocampal network activity and behaviour. These findings provide experimental evidence that maternal cell transfer can influence neuroimmune development in mice. However, they do not establish comparable functional effects in the human foetal brain or demonstrate that maternal psychological stress modifies this cellular process.

Maternal psychological distress has been associated in some studies with variation in maternal HPA-axis activity and inflammatory profiles, although the direction and consistency of these associations differ across cohorts (74–76). Maternal immune and inflammatory conditions may also influence placental immune regulation, glucocorticoid metabolism, and foetal immune-system development (8, 41, 72). Separately, microchimeric cells have been implicated in immune regulation and tissue-specific functions, and maternal microchimeric cells influence neuroimmune development in experimental mouse models (78, 79). Based on these distinct lines of evidence, one possible but currently untested hypothesis is that maternal endocrine or inflammatory conditions may modify the trafficking, persistence, phenotype, or functional activity of maternal microchimeric cells. However, no available human study has demonstrated that maternal psychological stress alters maternal microchimerism or that microchimeric-cell variation mediates offspring neurodevelopmental outcomes.

The relevance of maternal microchimerism to human neurodevelopment therefore remains exploratory. Evidence that maternal cells enter foetal tissues and persist after birth establishes the occurrence of maternal microchimerism but does not by itself demonstrate developmental function. Current functional evidence regarding effects on microglia, synaptic refinement, neural-network maturation, and behaviour is derived primarily from mouse models (78, 79). Human investigation remains limited by the low abundance and heterogeneous phenotype of microchimeric cells, restricted access to foetal and paediatric tissues, technical challenges in distinguishing maternal cells from foetal or host cells, and the absence of longitudinal studies linking cellular measurements with developmental outcomes.

It remains unclear whether variation in maternal microchimerism contributes causally to offspring neurodevelopment, represents a biomarker of broader placental or immune processes, or reflects context-dependent adaptation during pregnancy. The functional consequences of maternal microchimerism may differ according to tissue distribution, developmental stage, maternal health status, and inflammatory environment. Future studies integrating placental and cord-blood sampling, maternal-cell-specific genotyping, single-cell or spatial profiling, immune phenotyping, and longitudinal neurodevelopmental assessment will be required to clarify these possibilities (78, 79).

Overall, maternal microchimerism should be considered an emerging cellular mechanism of maternal–offspring communication that may intersect with immune regulation and neurodevelopment, rather than an established component of the HPA-axis–cortisol pathway. Experimental studies indicate that maternal cells can exert functional effects on neuroimmune development in mice, whereas human studies currently demonstrate persistent cellular exchange but have not established developmental consequences (78, 79). A multilayered model of maternal–foetal communication may therefore include endocrine signalling, placental regulation, inflammatory and immune processes, and cellular trafficking. However, interactions among these systems remain largely hypothetical, and maternal microchimerism should presently be regarded as a complementary candidate mechanism requiring direct human validation rather than a confirmed mediator linking maternal psychological states with offspring neurodevelopment.

### 6.4 Maternal and infant microbiome as an emerging developmental modulator

Beyond endocrine, placental, immune-inflammatory, and cellular pathways, the maternal and early-life microbiome has been proposed as another potential component of the developmental environment. Maternal microbial communities and microbiota-derived metabolites may interact with immune regulation, metabolic processes, and neuroendocrine signalling; however, direct evidence linking microbiome-related changes with offspring neurodevelopmental outcomes remains limited (80, 81).

Kimmel et al. (80) proposed the maternal microbiome as a conceptual framework connecting prenatal stress exposure, maternal physiological adaptation, microbial metabolites, immune signalling, and potential offspring psychiatric vulnerability. However, this framework does not establish the maternal microbiome as a validated biomarker or demonstrated mediator of developmental outcomes. Similarly, Yeramilli et al. (81) reviewed evidence suggesting that prenatal stress may be associated with alterations in maternal or offspring microbiota composition and microbial metabolite profiles, with potential implications for immune and neurodevelopmental processes. Nevertheless, much of the available evidence derives from animal models or small and heterogeneous human studies, and no consistent microbiome signature of prenatal psychological stress has been identified.

Eckermann et al. (82) conducted a longitudinal study examining maternal depressive, anxiety, and stress symptoms, maternal hair cortisol and cortisone concentrations, and maternal and infant gut microbiota across pregnancy and early postnatal life. Maternal stool samples were collected at 18 and 32 gestational weeks and at 8 months postpartum, while infant stool samples were collected at 2, 6, and 12 weeks and at 8 months. Associations between psychological or endocrine stress indicators and microbiota characteristics varied according to the exposure measure and sampling time and were not consistent across maternal and infant samples. Some maternal stress-related measures were associated with differences in infant microbiota features; however, no generalized microbiome pattern corresponding to prenatal stress was identified (82). Importantly, the study did not assess placental glucocorticoid metabolism or long-term neurodevelopmental outcomes and therefore could not determine whether microbiome variation mediated associations between maternal psychological distress, cortisol regulation, and offspring development.

Current evidence supports the possibility that maternal and early infant microbiota contribute to a broader biological context in which endocrine, immune, metabolic, and developmental processes interact. However, the relationships among microbiome composition, microbial metabolites, HPA-axis regulation, placental glucocorticoid metabolism, immune signalling, and offspring neurodevelopment have not yet been demonstrated in integrated longitudinal human studies. Therefore, microbiome-related mechanisms should currently be regarded as emerging, context-dependent candidate pathways rather than established regulators of glucocorticoid signalling or confirmed mediators of developmental outcomes (80–82).

Together with endocrine, placental, immune-inflammatory, and cellular pathways, microbiome-related processes further support a multilayered model of maternal–offspring communication. Future studies integrating longitudinal microbiome profiling, microbial metabolomics, maternal endocrine measurements, placental functional analyses, immune phenotyping, and long-term neurodevelopmental follow-up will be required to determine whether microbiome-related alterations represent causal mechanisms, biological mediators, or markers of broader maternal–offspring physiological adaptation.

### 6.5 Heterogeneity across studies and methodological considerations

First, maternal psychological exposures are defined inconsistently across studies. Perceived stress, depressive symptoms, anxiety symptoms, clinically diagnosed psychiatric disorders, adverse life events, and longitudinal symptom trajectories represent related but non-equivalent constructs. These measures differ in severity, duration, timing, clinical significance, and reliance on subjective assessment. Moreover, they are not consistently associated with the same patterns of maternal cortisol regulation. Therefore, combining these exposures under the broad category of “prenatal stress” may obscure exposure-specific associations and contribute to apparent inconsistencies across studies (1, 4, 10).

Second, developmental timing represents a major source of heterogeneity. Maternal cortisol secretion, placental CRH signalling, placental glucocorticoid metabolism, and foetal HPA-axis maturation undergo substantial changes across gestation. Therefore, cortisol concentrations measured during early pregnancy are not biologically equivalent to measurements obtained near delivery. Similarly, maternal serum, saliva, hair, nail, placental tissue, and cord blood reflect different biological compartments and temporal windows and should not be interpreted as interchangeable indicators of a single exposure process (16, 18, 24).

Placental studies illustrate these complexities. Huebner et al. (12) examined placental glucocorticoid metabolism using samples from different placental regions obtained after spontaneous vaginal delivery or caesarean delivery. The placental cortisol/cortisone ratio correlated with direct microsomal glucocorticoid-conversion capacity, whereas associations involving HSD11B2 mRNA varied according to delivery context. Placental CRH, cortisol, and cortisone concentrations were also influenced by labour status. These findings demonstrate that gene expression, steroid concentrations, metabolite ratios, and enzymatic activity represent distinct biological layers rather than equivalent measures. However, because the sample size was small and delivery characteristics were not experimentally manipulated, the study cannot determine which clinical factors accounted for the observed differences (12).

Third, glucocorticoid biomarkers differ substantially in biological interpretation and methodological sensitivity. Serum and salivary cortisol primarily reflect short-term circulating glucocorticoid dynamics and are influenced by sampling time, acute conditions, and circadian variation. Hair and nail glucocorticoids provide estimates of longer-term accumulation but may be affected by biological characteristics, sampling procedures, cosmetic treatment, storage conditions, extraction methods, and assay approaches. Cord blood, neonatal hair, and placental tissue represent different combinations of maternal, placental, foetal, labour-related, and early postnatal influences. Therefore, these biological matrices should not be considered interchangeable measurements of one continuous glucocorticoid exposure trajectory (12, 24, 26, 48).

Fourth, heterogeneity also arises from differences in covariate assessment and statistical modelling. Maternal psychiatric history, medication exposure, socioeconomic conditions, nutrition, metabolic health, obstetric complications, gestational age, parity, and delivery characteristics may influence both maternal biomarkers and offspring outcomes. Following birth, maternal mental health, caregiving environment, feeding, sleep, illness, socioeconomic conditions, and familial genetic factors may further modify developmental trajectories. However, inappropriate adjustment may introduce either residual confounding or overadjustment of variables that lie on potential causal pathways.

For example, Lamadé et al. (73) found that cord-blood BDNF concentrations were associated with both maternal depressive symptoms and lower socioeconomic status, illustrating the difficulty of separating correlated psychosocial exposures. The study did not establish whether socioeconomic conditions, maternal mood, or other biological factors represented the primary driver, nor whether BDNF mediated later developmental outcomes (73). Karl et al. (48) reported that maternal prenatal depressive symptoms remained associated with neonatal hair cortisol after adjustment for gestational age, delivery mode, parity, sample storage time, and pregnancy complications. Although persistence after adjustment supports an association, it does not exclude unmeasured confounding or demonstrate causality (48).

Fifth, foetal sex may contribute to biological heterogeneity, but current evidence does not support a universal male- or female-specific vulnerability model. Sex-related differences have been reported in placental cytokine expression, maternal inflammatory and cortisol-related associations, glucocorticoid metabolism, and offspring developmental outcomes. However, the direction and magnitude of effects vary according to exposure type, developmental stage, and experimental model (14, 27, 29, 32, 41). Future studies should prespecify and formally test exposure-by-sex interactions. Sex-stratified analyses may provide useful information when adequately powered, but differences in statistical significance between male and female subgroups do not by themselves demonstrate a true interaction.

Sixth, interpretation of maternal cortisol requires consideration of normal pregnancy physiology. Placental CRH secretion and maternal cortisol concentrations increase progressively across gestation as part of normal endocrine adaptation supporting foetal maturation and preparation for parturition. Therefore, higher cortisol concentrations during pregnancy cannot automatically be interpreted as evidence of pathological stress activation (1, 16).

Importantly, psychological and endocrine measures represent complementary but non-equivalent dimensions of maternal–foetal physiology. Psychological questionnaires assess perceived stress, mood symptoms, anxiety, or clinical symptom burden, whereas cortisol measurements reflect endocrine activity within specific biological contexts. Some studies demonstrate associations between psychological distress and cortisol regulation, whereas others report weak, null, timing-dependent, or directionally inconsistent findings (4, 23, 25). Neither psychological symptoms nor cortisol concentrations alone fully capture the complexity of maternal HPA-axis regulation.

Additional methodological challenges arise from placental molecular analyses. Placental tissue is usually obtained at delivery, whereas many exposures of interest occur months earlier. Findings may differ according to placental compartment, sampling location, cellular composition, labour status, and analytical approach. DNA methylation, gene expression, protein abundance, enzymatic activity, and cortisol/cortisone ratios represent related but distinct biological processes. Small sample sizes and multiple comparisons involving genes, CpG sites, cytokines, tissue regions, or sex interactions may further contribute to imprecise or non-replicable findings. These limitations are particularly relevant when molecular measurements obtained at delivery are interpreted as evidence of mechanisms operating during earlier developmental windows (12, 13, 22).

Overall, heterogeneity across this field reflects both genuine developmental complexity and methodological differences in exposure definition, gestational timing, biological matrices, placental assessment, outcome age, covariate selection, and analytical approaches. Future studies should integrate repeated psychological and endocrine measurements, functional placental assessments, clearly defined developmental windows, prespecified sex-interaction analyses, postnatal environmental factors, and longitudinal neurodevelopmental follow-up. Such approaches will be essential to distinguish transient physiological adaptation, persistent biological differences, correlated biomarkers, and causal developmental mechanisms (10, 39, 46).

## 7 Synthetic glucocorticoid exposure: a distinct clinical context and implications for developmental adaptation

Unlike variation in endogenous maternal cortisol and HPA-axis activity, antenatal synthetic glucocorticoid exposure represents an exogenous and pharmacologically defined alteration of foetal glucocorticoid signalling. Dexamethasone and betamethasone can cross the placenta and activate glucocorticoid receptors in foetal tissues; therefore, their biological effects may differ from physiological maternal cortisol exposure in terms of timing, magnitude, duration, and tissue distribution. In pregnancies at risk of preterm birth, antenatal corticosteroids are widely used to accelerate foetal maturation and reduce neonatal complications (83). However, because synthetic glucocorticoids may also influence foetal and neonatal HPA-axis regulation, their potential longer-term developmental associations require continued evaluation (84).

Antenatal corticosteroid therapy remains an important clinical intervention when preterm birth is anticipated. Repeat courses may provide additional short-term neonatal respiratory benefits in selected circumstances, although uncertainty remains regarding optimal regimens and longer-term outcomes (83, 85). Navalón et al. (86) followed 110 mother–child dyads after threatened preterm labour and compared children exposed to an initial corticosteroid course alone with those whose mothers received at least one rescue dose. At 30 months, children exposed to rescue doses showed higher salivary cortisol concentrations and lower problem-solving scores, with both outcomes showing variation according to the number of rescue doses received. However, rescue treatment was not randomly assigned, and additional corticosteroid exposure was closely linked to persistent risk of preterm birth and related clinical characteristics. Therefore, these findings indicate an association and possible dose-related pattern but cannot establish that rescue corticosteroid exposure directly caused altered HPA-axis regulation or developmental differences (86).

Importantly, these findings should not be interpreted as challenging the established neonatal benefits of appropriately indicated antenatal corticosteroid therapy. Rather, they highlight the need to clarify the long-term developmental consequences of repeated exposure and to optimise treatment strategies by balancing short-term neonatal benefits against potential later effects (83, 85).

Experimental studies provide mechanistic evidence that prenatal synthetic glucocorticoid exposure can produce persistent, organ-specific developmental alterations. Xiao et al. (87) used a prenatal dexamethasone rat model combined with *in-vitro* hepatocyte experiments and limited neonatal human validation data. Prenatal dexamethasone exposure was associated with foetal liver developmental abnormalities and later metabolic phenotypes in male offspring rats, including insulin resistance. Mechanistic analyses suggested that reduced endogenous corticosterone and impaired hepatic IGF1 signalling involved alterations in GRα–SP1–P300 regulation and histone acetylation at the Igf1 promoter. However, the human validation component was limited and did not establish comparable long-term metabolic outcomes or causal mechanisms in humans (87).

Glucocorticoid signalling contributes to brain maturation, neuronal differentiation, and HPA-axis feedback development; therefore, it remains biologically plausible that antenatal synthetic glucocorticoid exposure may influence neurodevelopment through molecular and epigenetic mechanisms. Nevertheless, Xiao et al. investigated hepatic and metabolic development rather than brain outcomes, and the IGF1-related pathway should not be interpreted as an established neurodevelopmental mechanism. Extrapolation from experimental models to human pregnancy requires careful consideration of species differences, dose, gestational timing, treatment duration, glucocorticoid type, foetal sex, placental regulation, and the clinical circumstances leading to treatment (18, 84, 87).

Overall, antenatal synthetic glucocorticoid exposure represents a clinical context distinct from variation in endogenous maternal cortisol regulation. Appropriately indicated corticosteroid treatment provides important neonatal benefits when preterm birth is imminent. Current evidence suggests that repeated or rescue exposure may be associated with differences in later endocrine or developmental measures, whereas experimental studies demonstrate potential organ-specific developmental effects of prenatal dexamethasone exposure. However, available evidence does not demonstrate that clinically indicated antenatal corticosteroid treatment uniformly causes adverse neurodevelopmental, metabolic, or psychiatric outcomes in humans (86, 87).

The developmental consequences of synthetic glucocorticoid exposure are likely to depend on treatment indication, gestational timing, agent, dose, number of courses, maternal and placental context, foetal characteristics, and subsequent postnatal environment. Current human evidence remains insufficient to define a single causal developmental pathway or to infer long-term neuropsychiatric risk from available endocrine and experimental findings alone.

## 8 Potential interventions and modifiable factors influencing offspring development

Recent research has increasingly focused on potentially modifiable psychosocial, behavioural, nutritional, caregiving, metabolic, and environmental factors that may improve maternal health or modify developmental conditions associated with prenatal adversity (10, 15, 88). However, the available evidence varies considerably in strength, ranging from randomized psychosocial and dietary interventions to observational studies of caregiving environments, maternal endocrine states, and environmental exposures.

Few studies have directly examined whether intervention-related changes in maternal HPA-axis regulation contribute to improvements in offspring development. Existing interventions have demonstrated benefits for maternal psychological symptoms, selected endocrine markers, and, in some cases, early infant developmental outcomes; however, current evidence does not establish normalization of maternal HPA-axis activity as the mechanism underlying these effects (10, 89, 90). Therefore, interventions should be considered multidimensional strategies that may influence interacting psychological, endocrine, immune, metabolic, placental, and postnatal pathways rather than as established HPA-axis-targeted therapies.

**Figure 2:**
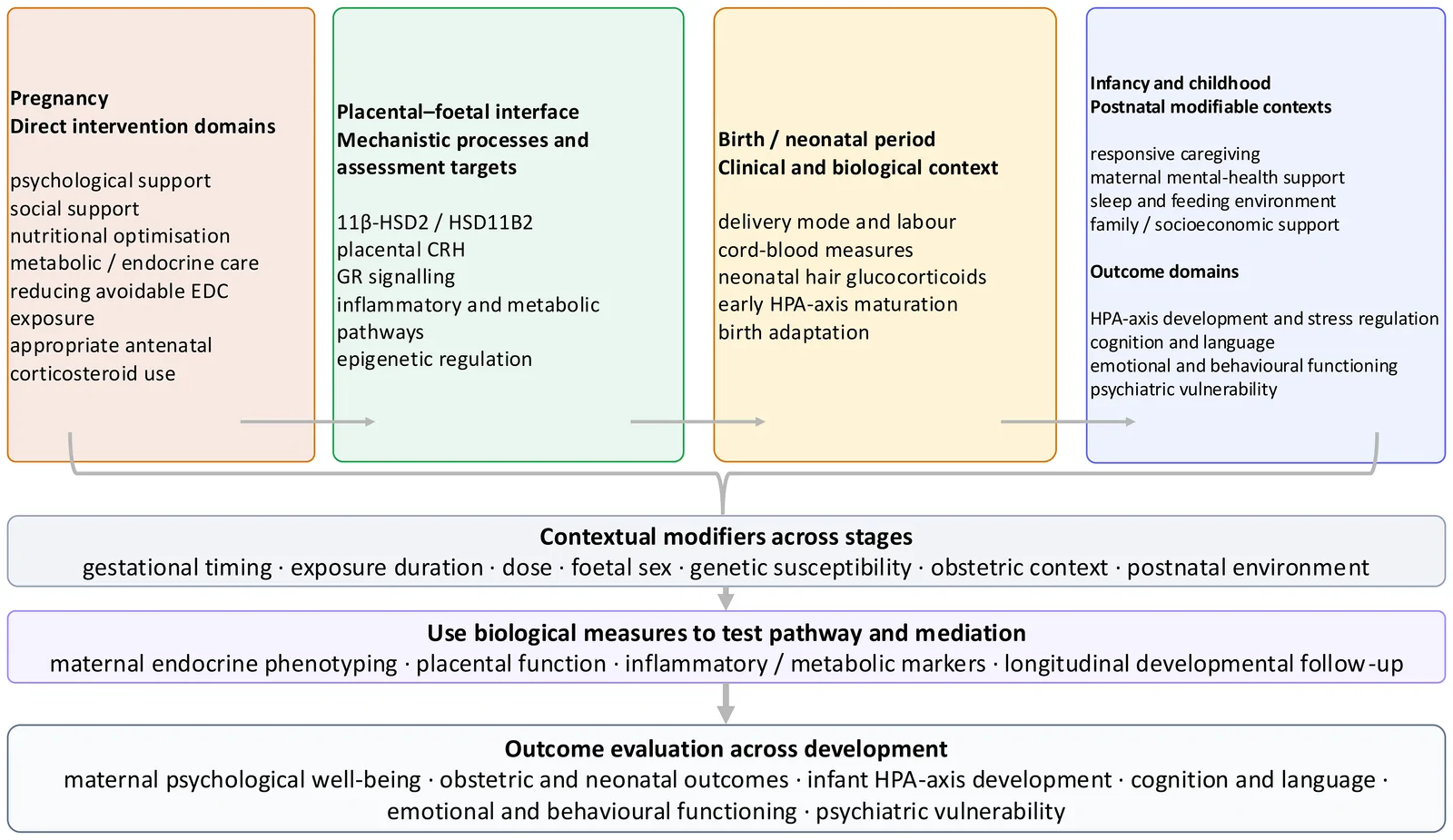
Potentially modifiable domains and intervention windows across development. The figure summarises potentially modifiable domains and clinically relevant contexts across pregnancy, the placental–foetal interface, birth and the neonatal period, and infancy and childhood. Pregnancy-related intervention domains include psychological and social support, nutritional optimisation, management of metabolic or endocrine conditions, reduction of avoidable endocrine-disrupting chemical exposure, and appropriate antenatal corticosteroid use. Placental glucocorticoid regulation, inflammatory and metabolic pathways, and epigenetic processes represent candidate mechanisms and assessment targets rather than established treatment targets. Interpretation across developmental stages should consider exposure timing, duration and dose, foetal sex, genetic susceptibility, obstetric context, and the postnatal environment. Biological measures should be used to test proposed pathways and mediation, alongside evaluation of maternal, obstetric, neonatal, neuroendocrine, cognitive, language, emotional, behavioural, and psychiatric outcomes.

**Figure 2** summarises potentially modifiable domains and intervention windows across pregnancy, the placental–foetal interface, birth and the neonatal period, and infancy and childhood.

### 8.1 Psychosocial and behavioural interventions

Psychosocial and behavioural interventions represent important approaches for reducing prenatal stress, anxiety, and depressive symptoms and improving maternal psychological adaptation during the perinatal period. These interventions primarily target maternal well-being and social functioning rather than defined HPA-axis abnormalities. Because prenatal psychological distress may influence offspring development through interacting endocrine, immune-inflammatory, placental, behavioural, and postnatal pathways, potential developmental benefits of these interventions are unlikely to be attributable solely to cortisol regulation (2, 10, 15). Evidence that psychosocial interventions improve offspring outcomes through maternal HPA-axis modulation remains limited.

Randomized intervention studies provide preliminary evidence that psychosocial interventions may influence selected HPA-axis-related biomarkers, although findings are not consistent and causal mediation has not been established. Puertas-González et al. conducted a randomized controlled trial of 48 pregnant women and reported that a stress-management cognitive behavioural intervention reduced maternal psychological symptoms, prevented increases in maternal hair cortisol observed in controls, and was associated with lower neonatal hair cortisol and higher infant cognitive and motor scores at 6 months (90). However, the small sample size and lack of mediation analysis prevent conclusions that cortisol changes mediated developmental differences. In a separate randomized intervention among women with prenatal depression in rural Pakistan, Baranov et al. reported lower maternal hair cortisol and cortisone and higher maternal and infant DHEA concentrations after intervention, whereas infant cortisol and cortisone did not differ between groups (89). These findings suggest that psychosocial interventions may influence selected endocrine measures but do not support a uniform HPA-axis normalization pathway across mothers and offspring.

Beyond prenatal interventions, the postnatal caregiving environment represents an additional modifiable context that may influence developmental trajectories following prenatal adversity. Maternal sensitivity, supportive caregiving, social resources, and socioeconomic conditions have been proposed as potential buffering factors, although human evidence remains largely observational (15). Within a developmental-cascade framework (91), Rinne et al. found that preconception and prenatal stress were associated with child temperament characteristics at 4 years, while more responsive parenting was associated with fewer behavioural problems at 5 years. However, these findings represent independent postnatal associations and do not demonstrate that parenting reverses or mediates prenatal stress-related effects (92).

Social relationships may also influence maternal psychological and endocrine states, although effects are context dependent. In a cohort of 107 pregnant Latina women, Fox et al. reported that favourable relationships with maternal grandmothers were associated with better maternal mental health and lower morning urinary cortisol, whereas paternal grandmother relationships showed mental-health benefits but higher urinary cortisol concentrations (93). These findings indicate that social support does not uniformly correspond to reduced cortisol and do not establish effects on HPA-axis regulation or offspring development.

Overall, current evidence supports psychosocial interventions and supportive relational environments as effective approaches for improving maternal psychological well-being, with some evidence of associated changes in endocrine biomarkers (89, 90, 93). However, neither cortisol alteration nor HPA-axis normalization has been established as the primary mechanism underlying offspring developmental benefits. Future studies should incorporate adequately powered randomized designs, repeated endocrine assessments, mediation analyses, placental biomarkers where feasible, evaluation of postnatal environments, and long-term developmental follow-up.

### 8.2 Nutrition, maternal metabolic health and early-life developmental environment

Maternal nutritional status may influence metabolic and inflammatory physiology, placental adaptation, and the developmental environment during pregnancy. Experimental evidence suggests that nutrient imbalance can interact with glucocorticoid-related pathways, although effects depend on nutrient type, exposure timing, tissue, and species (9, 30, 40). However, direct human evidence that nutritional interventions improve offspring neurodevelopment through modification of maternal HPA-axis activity or placental glucocorticoid regulation remains limited.

The IMPACT-BCN randomized controlled trial examined a Mediterranean-diet intervention among 1,221 pregnant women at increased risk of delivering a small-for-gestational-age infant. Participants were allocated at 19–23 weeks of gestation to a Mediterranean diet, mindfulness-based stress reduction, or usual care. The Mediterranean-diet intervention was associated with reduced perceived stress and anxiety symptoms and improved sleep-related outcomes during pregnancy (94). These findings support potential maternal psychological and well-being benefits but do not establish that effects were mediated by HPA-axis regulation or translated into long-term offspring developmental advantages.

Animal studies indicate that specific nutritional exposures may influence offspring endocrine and metabolic phenotypes, although these findings should not be interpreted as evidence for placental glucocorticoid-mediated neurodevelopmental programming. In rats, Nouchi et al. reported that maternal high-fructose corn-syrup intake during gestation and lactation was associated with increased corticosterone levels in male offspring and reduced renal 11β-HSD2 activity through a potential miR-27a–Hsd11b2 pathway (95). However, this study examined postnatal renal glucocorticoid metabolism rather than placental glucocorticoid regulation. Similarly, Coker et al. demonstrated timing- and sex-dependent effects of maternal vitamin C restriction on offspring growth and metabolic outcomes in guinea pigs (96). These findings highlight the importance of developmental timing and biological context but do not establish an HPA-axis-mediated mechanism in humans.

Early postnatal nutrition may also contribute to the developmental endocrine environment. Human milk contains cortisol and cortisone, with concentrations influenced by lactation stage, circadian variation, and maternal–infant characteristics (97–99). Hollanders et al. reported associations between breast-milk glucocorticoids and infant salivary cortisol rhythms at 1 month, although these relationships may reflect either endocrine signalling or broader mother–infant physiological synchrony (36). In contrast, Toorop et al. found no consistent association between breast-milk glucocorticoid rhythmicity and infant behaviour or sleep outcomes at 3 months (100). Thus, milk glucocorticoids represent a potential component of the early postnatal endocrine environment, but their contribution to infant HPA-axis maturation and neurodevelopment remains uncertain.

Overall, current evidence supports nutrition and early-life feeding environments as potentially modifiable contributors to maternal and offspring health. However, available data do not demonstrate that nutritional interventions improve offspring neurodevelopment through maternal HPA-axis modulation, placental glucocorticoid metabolism, or milk glucocorticoid signalling. Future studies should integrate dietary assessment with maternal endocrine and metabolic measures, placental functional markers, postnatal feeding exposures, and longitudinal developmental outcomes.

### 8.3 Environmental exposures, endocrine health and risk modification

Maternal metabolic and endocrine conditions may contribute to variation in the prenatal developmental environment through alterations in steroid metabolism, inflammation, and metabolic physiology. Gerszi et al. identified first-trimester differences in cortisol, cortisone, steroid metabolites, and oxidative–metabolic markers among women who later developed gestational diabetes mellitus, while Ondřejíková et al. reported altered maternal steroid profiles in pregnancies complicated by gestational diabetes (101, 102). These findings indicate associations between maternal metabolic disorders and glucocorticoid-related physiology; however, they do not establish altered placental 11β-HSD2 function, increased foetal glucocorticoid exposure, or HPA-axis-mediated effects on offspring development. Evidence that optimisation of maternal endocrine health improves offspring neurodevelopment specifically through glucocorticoid pathways remains limited.

Environmental endocrine-disrupting chemical (EDC) exposures represent another potentially modifiable component of the prenatal environment. In a systematic review of 22 epidemiological studies, Pande et al. examined prenatal exposure to phthalates, PFAS, phenols, parabens, and related chemicals in relation to maternal and offspring glucocorticoids and placental CRH. Although several studies reported associations with HPA-axis-related biomarkers or sex-specific effects, findings varied substantially across chemical classes, biological matrices, developmental periods, and outcomes (88). Current evidence therefore suggests possible endocrine vulnerability to environmental exposures but does not support a consistent exposure–HPA-axis response pattern.

Dreyer et al. examined first-trimester PFAS exposure in relation to maternal cortisol and cortisone measures later in pregnancy. Higher PFOS exposure was associated with lower third-trimester urinary cortisone and a higher cortisol-to-cortisone ratio, findings potentially consistent with altered glucocorticoid metabolism (103). However, placental 11β-HSD2 activity, foetal glucocorticoid exposure, and offspring developmental outcomes were not assessed; therefore, these findings cannot establish disruption of the placental glucocorticoid barrier or developmental consequences.

Maternal age and related sociodemographic and obstetric factors should be considered contextual variables rather than direct intervention targets. Moreno-Giménez et al. reported associations between maternal age and infant personal–social development and temperament-related outcomes after adjustment for paternal age, education, parity, social support, maternal cortisol, and psychological symptoms (104). However, such associations likely reflect multiple correlated biological, obstetric, and social factors rather than a single endocrine mechanism.

Overall, maternal metabolic conditions and environmental exposures represent important components of the prenatal developmental context. However, current evidence does not demonstrate that modifying these factors improves offspring neurodevelopment through maternal HPA-axis regulation or placental glucocorticoid pathways. Future studies should incorporate prospective exposure assessment, repeated endocrine measurements across biological matrices, placental functional analyses, and long-term offspring follow-up (6, 88).

### 8.4 Summary and future perspectives

Overall, evidence regarding modifiable factors and interventions relevant to maternal HPA-axis regulation and offspring development remains limited and heterogeneous. The strongest intervention evidence currently comes from psychosocial and dietary randomized trials. These interventions have demonstrated improvements in maternal psychological well-being, selected endocrine biomarkers, and, in one small trial, early infant developmental outcomes. However, available studies do not establish that cortisol changes mediate these benefits or that HPA-axis normalization represents the mechanism responsible for improved offspring development (89, 90, 94).

Evidence for caregiving environments, maternal metabolic conditions, nutritional mechanisms, and environmental exposures remains largely observational or experimental. Supportive caregiving and responsive parenting may contribute to more favourable developmental trajectories following prenatal adversity, while metabolic and environmental studies identify potential biological pathways involving endocrine, immune, and placental processes. However, these findings currently define candidate mechanisms rather than proven intervention targets or causal pathways (15, 36, 88, 92, 95, 96).

Current evidence therefore does not support reducing maternal cortisol as a standalone preventive strategy. Cortisol variation, placental glucocorticoid metabolism, inflammatory markers, and offspring HPA-axis-related biomarkers should instead be considered indicators within a broader maternal–placental–offspring developmental system. Interventions should be evaluated primarily according to demonstrated maternal, obstetric, neonatal, and developmental benefits, while biological measures are used to clarify underlying mechanisms (10, 89, 90).

Future research should apply designs appropriate to each intervention domain. Randomized controlled trials of psychosocial, behavioural, nutritional, and caregiving interventions should incorporate repeated endocrine assessments, placental biomarkers where feasible, prespecified mediation analyses, intervention fidelity assessment, and long-term offspring follow-up. For non-randomizable exposures, including environmental chemicals, maternal age, parity, and metabolic or obstetric conditions, prospective cohorts, natural experiments, and causal-inference approaches are required. Across study designs, longitudinal integration of maternal psychological measures, endocrine profiles, placental function, neonatal biomarkers, and childhood developmental outcomes will be essential to distinguish causal mechanisms from correlated biomarkers and contextual factors (10, 88).

## 9 Future directions

Current evidence indicates that maternal HPA-axis regulation and glucocorticoid signalling are important components of the prenatal developmental environment, but offspring trajectories cannot be explained by a single hormonal pathway. Maternal psychological state, placental endocrine regulation, immune-inflammatory and metabolic processes, genetic and epigenetic susceptibility, and postnatal experiences likely interact across developmental stages (2, 10, 39, 46). Future research should therefore move beyond the “single-hormone exposure” model towards a dynamic maternal–placental–offspring regulatory-network framework that considers developmental timing and biological context (1, 10, 19, 40).

A major priority is improving causal inference. Most human studies remain observational, limiting the ability to distinguish causal mechanisms from biological correlates, adaptive responses, or consequences of maternal conditions (2, 10, 46). Progress will require integration of large longitudinal cohorts with repeated exposure and outcome measurements, negative-control approaches, sibling-comparison designs, natural experiments, and mechanistically informative interventions. Genetically informed methods, including Mendelian randomisation where appropriate, may provide additional evidence but require careful consideration of maternal and foetal genetic contributions.

Improved characterization of HPA-axis physiology is also needed. Single cortisol measurements cannot adequately capture diurnal regulation, cumulative exposure, stress reactivity, feedback sensitivity, or tissue-specific glucocorticoid metabolism (4, 24, 26). Future studies should combine repeated endocrine assessments, including serum or salivary cortisol profiles, hair or nail glucocorticoids, and functional measures of HPA-axis regulation where feasible.

Placental biomarkers may provide additional mechanistic insight into glucocorticoid metabolism, receptor signalling, immune regulation, and molecular adaptation. However, placental samples obtained at delivery represent a specific late-pregnancy tissue state and should not be interpreted as direct measures of foetal exposure throughout gestation. Future studies should integrate placental DNA methylation, gene expression, protein abundance, enzymatic activity, and steroid measurements with maternal and offspring biomarkers while accounting for tissue compartment, sampling location, labour status, and clinical context (12, 13, 22, 40).

Intervention studies should also determine not only whether psychosocial, behavioural, nutritional, or caregiving approaches improve maternal and offspring outcomes, but also through which biological and environmental pathways these effects occur. Well-designed trials should incorporate mediation analyses, repeated endocrine and inflammatory assessments, placental measures where feasible, and long-term developmental follow-up. For non-randomisable exposures, prospective cohorts and causal-inference approaches remain essential. Importantly, reductions in maternal cortisol should not be considered either a necessary or sufficient surrogate for developmental benefit (10, 88–90, 94).

Future research should therefore address more specific questions: which patterns of maternal HPA-axis regulation are associated with developmental variation, during which developmental windows, through which maternal–placental–offspring pathways, and under what biological and environmental conditions these associations persist or change. Integrating longitudinal cohorts, advanced endocrine phenotyping, functional placental biology, environmental exposure assessment, genetics, epigenetics, and multi-omics approaches may enable more precise models of developmental adaptation and vulnerability (10, 39). Physiological glucocorticoid signalling remains essential for pregnancy and foetal maturation and should not be equated with pathology or considered an indiscriminate therapeutic target (16, 18).

## 10 Conclusion

Perinatal HPA-axis regulation represents an important component of the maternal–placental–foetal system through which maternal physiological and psychological conditions may contribute to offspring developmental trajectories. Current evidence indicates that maternal psychological distress, endogenous glucocorticoid regulation, placental endocrine and immune processes, and antenatal synthetic glucocorticoid exposure may interact across multiple developmental pathways. These associations are shaped by exposure timing, placental regulation, foetal susceptibility, maternal health, and postnatal environmental influences rather than by maternal cortisol alone (2, 10, 40).

Associations between maternal HPA-axis-related measures and offspring neuroendocrine, cognitive, language, emotional, behavioural, and psychiatric outcomes have been reported, but findings remain heterogeneous. Differences in psychological exposure definitions, gestational timing, biological matrices, placental sampling approaches, assay methods, offspring developmental stage, and covariate adjustment limit direct comparison and causal interpretation. Current evidence therefore supports a context-dependent developmental adaptation framework rather than a simple linear pathway from maternal distress to cortisol alteration and later neuropsychiatric disorders (4, 22, 24, 46).

Psychosocial and dietary interventions have shown benefits for selected maternal psychological and physiological outcomes, while caregiving environment, metabolic health, and environmental exposures represent additional modifiable developmental contexts. However, sustained offspring developmental benefits and the biological pathways underlying these effects remain insufficiently established. Future research should determine when HPA-axis-related measures provide additional mechanistic or predictive value beyond broader maternal, placental, foetal, and postnatal factors. Longitudinal and mechanistically informed studies integrating endocrine, placental, immune, metabolic, genetic, and environmental assessments will be essential to distinguish causal pathways from adaptive responses and correlated biomarkers.
